# *Mycobacterium abscessus* Induces CD18-Dependent Reactive Oxygen Species Production in Human Mast Cells

**DOI:** 10.3390/cells15151385

**Published:** 2026-07-31

**Authors:** Ilse Mendoza-Trujillo, Patricia Diez-Echave, Chiara Tontini, Rajia Bahri, Jennifer S. Cavet, Lydia Tabernero, Silvia Bulfone-Paus

**Affiliations:** 1 Lydia Becker Institute of Immunology and Inflammation, School of Biological Sciences, Faculty of Biology, Medicine and Health, University of Manchester, Manchester M13 9PL, UK; ilse.mendozatrujillo@postgrad.manchester.ac.uk (I.M.-T.); chiara.tontini@manchester.ac.uk (C.T.); rajia.bahri-2@manchester.ac.uk (R.B.); jennifer.s.cavet@manchester.ac.uk (J.S.C.);; 2 Allergy Research Group, Instituto de Investigación Biomédica de Málaga y Plataforma en Nanomedicina-IBIMA Plataforma BIONAND, 29590 Málaga, Spain; pdiezechave@gmail.com

**Keywords:** mast cells, *Mycobacterium abscessus*, reactive oxygen species, CD18

## Abstract

*Mycobacterium abscessus* (*Mab*) is an opportunistic pathogen that causes severe infections, especially in immunocompromised individuals. The role of human mast cells (hMCs) during *Mab* infection remains poorly characterised. This study examines early interactions between *Mab* and hMCs, evaluating MC viability, degranulation, reactive oxygen species (ROS) production, cytokine secretion, and receptor expression following *Mab* exposure. MCs interacted with both smooth (ATCC 19977) and rough (clinical isolate) *Mab* morphotypes. Infection did not induce MC degranulation. Instead, *Mab* primarily stimulated intracellular ROS generation, Granzyme B, and IL-1β production. CD18 and CD46 were necessary for hMC-*Mab* interactions, and CD18 deficiency significantly impacted ROS production. hMC cytokine priming with IL-33 or IFN-γ did not significantly affect CD18 expression, nor did it impact ROS production upon *Mab* infection. These findings demonstrate that MCs mount a rapid, receptor-dependent intracellular response to *Mab*, highlighting an early and previously underappreciated role of MCs in *Mab* infection.

## 1. Introduction

*Mycobacterium abscessus* (*Mab*) is a rapid-growing non-tuberculous mycobacterium (NTM) widely distributed in natural environments, including soil, water, and dust [1]. Although environmental in origin, *Mab* is an opportunistic pathogen capable of causing severe infections, particularly in immunocompromised individuals and patients with underlying conditions, such as cystic fibrosis [2]. *Mab* exhibits two distinct colony morphotypes, smooth and rough, resulting from mutations in genes affecting glycopeptidolipid (GPL) biosynthesis or transport (e.g., mmpL4, mps1, mps2, and gap). These mutations lead to the loss of surface GPLs, altering cell wall properties and host–pathogen interactions [3]. The smooth morphotype is characterised by biofilm formation and sliding motility [4]. It has been reported to impair macrophage antimicrobial functions by inhibiting apoptosis and preventing phagosomal acidification [5], thereby promoting intracellular survival and immune evasion. In contrast, the rough morphotype forms serpentine cords and bacterial aggregates and is associated with enhanced virulence and increased inflammatory responses [5].

Mast cells (MCs) are tissue-resident immune cells derived from CD34+/CD117+/CD13+ hematopoietic stem cell precursors in the bone marrow. After migrating through the bloodstream, they populate peripheral tissues such as the skin, lungs, and mucosal surfaces, where they complete their maturation [6]. However, recent evidence has shown that a subset of skin-resident MCs is yolk sac-derived and maintained independently of bone marrow cell progenitors [7]. Due to their strategic anatomical location and rapid responsiveness to environmental stimuli, MCs play a critical role in host defence against infection. Upon activation, they release a broad spectrum of pre-stored and *de novo* synthesised mediators, thereby contributing to immune cell recruitment and modulation of inflammatory responses [8,9,10]. Beyond mediator release, MCs possess phagocytic and antigen-presenting capabilities, further supporting their role in antimicrobial immunity [11,12].

In the context of mycobacterial infections, MCs have been shown to recognise *M. tuberculosis* (*Mtb*) through pattern recognition receptors (PRRs), including TLR2 and the GPI-anchored surface molecule CD48 [13,14]. MCs can phagocytose *Mtb* as well as other mycobacteria, such as *M. bovis* (BCG) and the NTM *M. marinum* [13,15,16,17]. Recently, in *Mtb*-infected lungs, MCs localise to pneumonic regions, accumulate near granulomas, and are enriched in fibrotic lesions, suggesting a role in disease progression and tissue remodelling [18].

Despite growing evidence supporting the involvement of MCs in mycobacterial immunity, their role during NTM infection, particularly *Mab,* remains poorly understood. MCs are strategically located at barrier surfaces and act as early immune sentinels [9,19]. Therefore, elucidating their contribution to the host response against *Mab* is of particular interest, specifically the mechanisms governing human MCs (hMCs)-*Mab* interactions and the functional responses elicited upon pathogen recognition.

Therefore, this study aimed to investigate the role in the early immune response to *Mab*. Specifically, we examined hMC-*Mab* interactions using the reference strain ATCC 19977 and a clinical isolate, assessed MC effector responses, and explored the contribution of membrane receptors to intracellular signalling pathways.

## 2. Materials and Methods

### 2.1. Human Mast Cell Progenitor Isolation and Cell Culture

hMCs were generated as previously described [20]. Blood samples for cell cultures were obtained from the Manchester NHS blood centre after approval by the University of Manchester Research Ethics Committee (UREC ref. 2018-2696-5711, Approval Date: 25 April 2018). CD117-positive MC progenitors were isolated by immunomagnetic sorting (Miltenyi Biotec, Bergisch Gladbach, Germany) from peripheral blood mononuclear cells (PBMCs). hMCs were initially cultured for 2 weeks in StemSpan medium (STEMCELL Technologies, Vancouver, BC, Canada) supplemented with penicillin (100 U/mL), streptomycin (100 μg/mL), recombinant human IL-3 (10 ng/mL; PeproTech, Cranbury, NJ, USA), IL-6 (50 ng/mL; PeproTech, Cranbury, NJ, USA), and stem cell factor (SCF; 100 ng/mL; PeproTech, Cranbury, NJ, USA). Subsequently, cells were transferred to Iscove’s Modified Dulbecco’s Medium (IMDM) + GlutaMAX (Thermo Fisher Scientific, Waltham, MA, USA) and supplemented with penicillin (100 U/mL), streptomycin (100 μg/mL), β-mercaptoethanol (50 μmol/L), bovine serum albumin (BSA; 0.5%), insulin-transferrin-selenium (ITS; 1%; Invitrogen, Carlsbad, CA, USA), recombinant human IL-3 (10 ng/mL), IL-6 (50 ng/mL), and SCF (100 ng/mL) (Media I) for a further 14 days. Cells were changed to the above medium without IL-3 from week 4 onwards (Media II). hMCs were tested for purity and maturity after 8 weeks of culture, achieving a purity of >95% (Supplementary Figure S1). The THP-1 monocyte cellular line (ATCC, TIB-202) was grown and maintained in RPMI (Sigma-Aldrich, St. Louis, MO, USA) medium containing L-glutamine supplemented with fetal bovine serum 10% (FBS; Invitrogen, Waltham, MA, USA) at 37 °C in 5% CO_2_.

### 2.2. Mycobacterial Strains and Culture Conditions

The *Mycobacterium abscessus* (*Mab*) 19977 strain (smooth type) was obtained from the American Type Culture Collection (ATCC), and the *Mab* clinical isolate (rough type) was kindly provided by Dr. Jose Antonio Dominguez from the Institut d’Investigació en Ciències de la Salut Germans Trias i Pujol, Badalona, Spain (Supplementary Figure S2). Bacteria were grown in Middlebrook 7H9 broth (BD Biosciences, Franklin Lakes, NJ, USA) medium containing glycerol 0.2% (Fisher Scientific, Loughborough, UK), Tween 80 0.05% (Fisher Scientific, UK), and oleic albumin dextrose catalase 10% (OADC). Bacteria were harvested during the logarithmic growth phase by centrifugation at 2000× *g* for 15 min, washed two times with PBS, resuspended in Middlebrook 7H9 broth medium containing glycerol 15% and stored at −80 °C. At every step, bacterial cultures were passed 5 times through a 25-gauge syringe to disrupt clumps. After at least 3 days at −80 °C, aliquots were defrosted and colony forming units (CFUs) determined following plating on 7H11 agar (Sigma-Aldrich, St. Louis, MO, USA) containing glycerol 0.5% and OADC 10% to determine culture concentration (CFU/mL). CFUs were enumerated after 4 days of incubation at 37 °C. Infection assays were planned according to the concentrations previously determined.

### 2.3. Microscopy

WGA-labelled hMCs (0.2 × 10^5^ cells/mL) were placed in 8-well Ibidi chambers (Ibidi GmbH, Gräfelfing, Germany) in HBSS supplemented with SCF (100 ng/mL) for 30 min before infection at 37 °C and 5% CO_2_. *Mab* ATCC 19977 or the clinical isolate was added at a Multiplicity of Infection (MOI) of 10 (10 bacteria per hMC), and the chambers were placed on the microscope, pre-set at 37 °C and 5% CO_2_. The MOI used in this experiment was selected based on previous studies investigating the interaction between MCs and mycobacteria, including *Mtb* and BCG, using microscopy-based approaches [14,16]. All samples were protected from light during the experiment to prevent fluorophore degradation. Live cell imaging was performed using an Eclipse Ti inverted microscope (Nikon Instruments Inc.) with a 40×/0.45 SPlan Fluor objective, the Nikon filter sets for GFP and mCherry, and a pE-300 LED (CoolLED, Andover, UK) fluorescent light source. Imaging software NIS Elements AR.46.00.0. Point visiting was used to allow multiple positions to be imaged within the same time-course, and cells were maintained at 37 °C and 5% CO_2_. The images were acquired using a Retiga R6 (Q-Imaging, Surrey, BC, Canada) camera with a Z-axis optical spacing of 0.1 μm, and only the maximum-intensity projections are shown in the results. Image stacks were summed and projected onto a single plane, and the resulting image was analysed using Fiji [21]. hMC-*Mab* contact events were manually counted, yielding a total of 19 cells per field per experiment.

### 2.4. Cell and Bacteria Fluorescent Labelling

1 × 10^8^ *Mab* CFU were washed with PBS twice at 2000× *g* for 15 min, followed by incubation with Carboxyfluorescein N-succinimidyl ester (CFSE, 10 μM) (Sigma, St. Louis, MO, USA) for 1 h at room temperature. The bacterial pellet was washed twice with PBS and used for experiments.

hMCs (0.2 × 10^5^ cells/mL) were incubated with a solution of Wheat Germ Agglutinin conjugated with CF^®^568 dye (CF^®^568 WGA) 5.0 µg/mL in Hanks’ Balanced Salt Solution (HBSS) (Dilution: 1/100, Biotium, Fremont, CA, USA) for 10 min at 37 °C, protected from light. After incubation, cells were washed twice in HBSS and resuspended in Media II for further experiments.

### 2.5. Mab Internalization

0.3 × 10^6^ hMCs/well were seeded in 24-well plates and infected with *Mab* for 4 h with an MOI of 1 based on previous infection assays [5]. Cells were then washed three times with PBS without antibiotics to mechanically remove extracellular bacteria and incubated at 37 °C in 5% CO_2_. CFU counts were performed by adding 1% Triton to lyse the hMCs, and then 10-fold serial dilutions were plated on 7H11 plates. CFUs were enumerated after 3–5 days of incubation at 37 °C. THP-1 cells were differentiated into macrophages at a concentration of 0.5 × 10^6^ cells/mL in a flat bottom plate by incubating cells overnight with phorbol 12-myristate 13-acetate (PMA, 25 nM; Thermo Fisher Scientific, Waltham, MA, USA). Cells were washed and rested for 1 day in RPMI medium before infection with *Mab* as described for hMCs.

### 2.6. Flow Cytometry

hMCs were seeded into flat-bottom plates at a final concentration of 0.5 × 10^6^ cells/mL using Media II without antibiotics. *Mab* ATCC 19977 or the clinical isolate was added at MOIs of 5, 10, or 20 and incubated for 2 h. After stimulation, MCs were washed with PBS and stained for 10 min with Fc block (BioLegend, 1:100 dilution) and LIVE/DEAD™ Fixable Blue or near-IR dead cell dye (Life Technologies, 1:1000 dilution) in PBS to distinguish live and dead cells. For functional assays, anti-human CD63 (clone H5C6, 1:200 dilution, BioLegend, San Diego, CA, USA) and CD107a (clone H4A3, 1:200 dilution, BioLegend, San Diego, CA, USA) were added in FACS buffer for 20 min at 4 °C. For membrane receptor staining, the following antibodies were used at 1:200 dilution: CD54 (ICAM-1, clone HCD54), CD203c (clone NP4D6), CD48 (clone BJ40), CD53 (clone QA19A07, BioLegend), CD81 (TAPA1, clone 5A6, BioLegend), Siglec-5/14 (clone REA393), MRGPRX2 (clone K125H4), L1CAM (clone L1-OV198.5), Siglec-6 (clone 767329), Siglec-3/CD33 (clone WM53, Miltenyi Biotec), DAP-12 (clone REA900, Miltenyi Biotec), CD46 (clone TRA-2-10, BioLegend), CD18 (clone TS1/18, BioLegend), CD11c (clone B-ly6), CD11b (clone ICRF44), TLR2 (clone TLR2.1), and TLR4 (clone HTA125). Following staining, cells were washed and fixed with 4% paraformaldehyde in PBS for 20 min at 4 °C, then resuspended in FACS buffer. Fluorescence minus one (FMO) controls were used for gating, and compensation was performed using single-stained cells. Flow cytometric analyses were conducted on a BD LSR-II flow cytometer (BD Biosciences), measuring a minimum of 10,000 events per sample. Data were analysed using FlowJo software (version 10.8.1; BD Biosciences).

### 2.7. Detection of Reactive Oxygen Species (ROS) in hMCs

hMCs were washed three times and subsequently seeded in flat-bottom plates at a final concentration of 0.5 × 10^6^ cells/mL using antibiotic-free Media II. Cells were pre-loaded for 15 min with the cell-permeant ROS-sensitive probe dihydrorhodamine 123 (DHR 123; Thermo Fisher Scientific) at a final concentration of 1.33 μg/mL. Following dye loading, *Mab* ATCC 19977 or the clinical isolate was added at MOIs of 5, 10, or 20, and cells were incubated for 2 h at 37 °C with 5% CO_2_. At the 2-h time point, ROS production was measured either by flow cytometry or using an Infinite 200 PRO plate reader (excitation: 488 nm; emission: 530 nm). For flow cytometry analysis, samples were stained with a live/dead viability dye and analysed as previously described.

### 2.8. Antibody-Mediated Blocking of Surface Receptors

hMCs were incubated for 1 h at 37 °C with anti-human CD48 (clone BJ40, BioLegend), CD46 (clone TRA-2-10, BioLegend), CD18 (clone TS1/18, BioLegend), or isotype control (Mouse IgG1, κ, BioLegend) antibodies at a concentration of 5 µg/mL in Media II. After incubation, cells were washed twice with PBS to remove antibiotics and seeded at 0.5 × 10^6^ cells/mL. *Mab* ATCC 19977 or clinical isolate was added using an MOI of 10, which was selected based on its ability to induce the highest ROS response in preliminary experiments. Samples were processed for downstream analysis, including live-cell imaging and detection of reactive oxygen species.

### 2.9. Cellular Priming of hMCs with IL-33 or IFN-γ

For cytokine priming, hMCs at a concentration of 0.5 × 10^6^ cells/mL were incubated overnight with IL-33 (50 ng/mL; PeproTech) or IFN-γ (50 ng/mL; PeproTech) in Media II at 37 °C in 5% CO_2_. Following incubation, cells were washed to remove residual cytokines, and *Mab* was added at the appropriate MOI for subsequent analyses of receptor expression and ROS production.

### 2.10. Differential Gene Expression Analyses

Data for the differential gene expression of key genes involved in the anti-mycobacterial response (CD14, CD36, CD46, CD48, CD209, CLEC7A, FPR1, FPR2, ITGAM, ITGB2, MARCO, MRC1, MSR1, NOD1, NOD2, TLR2, TLR4) were extracted from a previously published dataset [22]. The dataset was generated by pre-incubating hMCs for 24 h with IL-33 (50 ng/mL), IFN-γ (50 ng/mL), or IL-6-depleted Medium II (unstimulated control), followed by mRNA extraction using commercially available kits (Qiagen, Hilden, Germany). Transcriptomic analysis was performed by the Genomics Core Technology Facility of The University of Manchester, and raw counts were further analysed using the R package DESeq2 [23].

### 2.11. Statistical Analysis

Statistical analyses were performed using GraphPad Prism 9 software (GraphPad Software, San Diego, CA, USA). Data normality was assessed using the Shapiro–Wilk test. For normally distributed data, unpaired *t*-tests or one-way ANOVA followed by Tukey’s multiple comparisons test were used, as appropriate. For non-normally distributed data, the Mann–Whitney test or Kruskal–Wallis test followed by Dunn’s multiple comparisons test was applied. A *p* value < 0.05 was considered statistically significant.

## 3. Results

### 3.1. Human Mast Cells Interact with Distinct Morphotypes of M. abscessus Exhibiting Limited Phagocytic Activity

Previous microscopy studies have shown that MCs actively interact with different mycobacteria species [13]: *Mtb* can adhere to the MC surface [15], while BCG briefly engages MCs, with MC-BCG contacts usually lasting less than one minute [24]. Based on this, we characterised and quantified hMCs-*Mab* interactions using two distinct *Mab* colony morphotypes [25,26], namely smooth (ATCC 19977) and rough (clinical isolate) *Mab* (Supplementary Figure S2). The number of hMC-*Mab* contacts occurring at the cell membrane or along cellular protrusions was manually quantified across three independent experiments (representative videos are included as Supplementary Videos S1 and S2).

**Figure 1:**
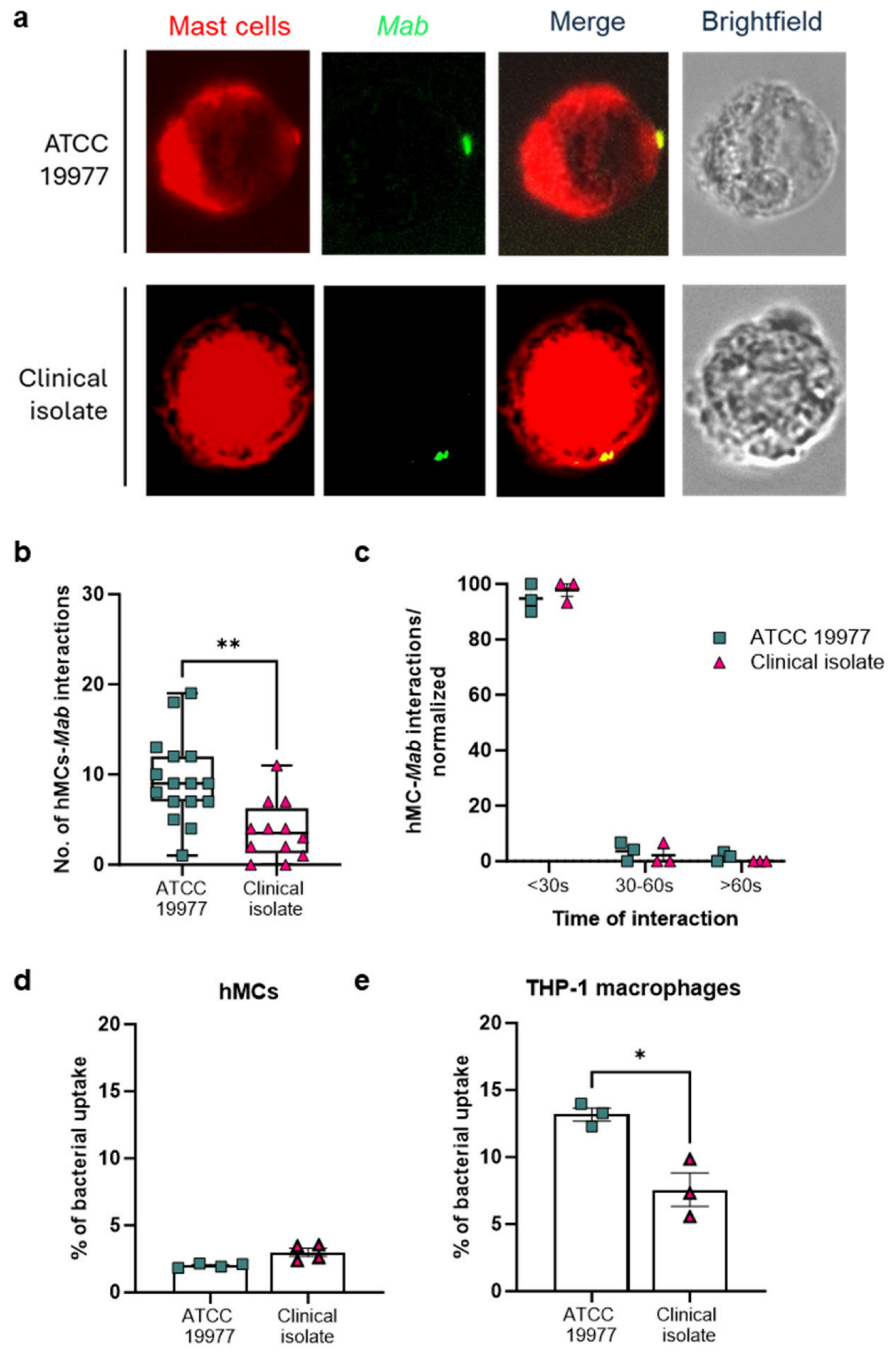
*Mab* interacts with hMCs without significant internalization. WGA-labelled hMCs (red) were exposed to *Mab* CFSE-labelled bacteria (green) at an MOI of 10. Live-cell imaging was performed for at least 30 min at 40× magnification at 37 °C and 5% CO_2_. (**a**) Micrograph showing hMC-*Mab* interaction using *Mab* ATCC 19977 or a clinical isolate. (**b**) The total number of interactions was counted manually for each cell for 30 min. The graph shows the distribution of hMC–*Mab* interactions at the single-cell level from one representative experiment and is presented for descriptive purposes. Later, (**c**) each interaction was classified according to the length of contact, normalising to the total number of interactions for each morphotype. The graph shows three independent experiments. A total of 19 cells per power field were analysed in each condition. Data were analysed using FIJI, and single contacts between bacilli at the MC membrane or their protrusions were considered an interaction. (**d**) hMCs or (**e**) THP–1-derived macrophages were infected for 4 h with the two different strains using an MOI of 1. After infection, samples were lysed, and intracellular bacterial CFU counts were determined. Data in (**d**) represents four independent experiments, each performed with three technical replicates (*n* = 4). Data in (**e**) shows one representative experiment from three independent experiments and are presented for descriptive purposes only. Data are expressed as means ± SEM. Statistical analysis was performed using (**d**) paired or (**e**) unpaired *t*-test. A *p* < 0.05 was considered statistically significant (** *p* < 0.01, * *p* < 0.05). Green squares represent the ATCC 19977 strain, while pink triangles represent the clinical isolate.

In our experimental model, both bacterial strains were observed interacting with the MC membrane (Figure 1a). The ATCC 19977 strain showed higher number of contacts compared to the clinical isolate (Figure 1b). No differences were observed in contact duration between *Mab* morphotypes, with approximately 90% of interactions lasting less than 30 s for both strains (Figure 1c).

MCs have previously been shown to internalise several mycobacterial species, including *Mtb*, BCG, and *M. marinum* [14,16,17]. Therefore, we next assessed whether hMCs internalise *Mab* and whether uptake differed between morphotypes.

hMCs were exposed to either ATCC 19977 or the clinical isolate and, after the removal of extracellular bacteria, internalisation was quantified by CFU counting. THP-1-derived macrophages were included as a positive control due to their established capacity to phagocytose *Mab* [5,27]. Because extracellular bacteria were removed without an antibiotic protection step, the CFU assay may overestimate intracellular bacterial counts due to strongly adherent extracellular bacteria.

hMCs internalised *Mab* at low levels, approximately 2–3% of the initial inoculum (Figure 1d). No significant difference was observed between ATCC 19977 and the clinical isolate (2% ± 0.1 and 3% ± 0.3, respectively). In contrast, THP-1-derived macrophages internalised higher levels of bacteria, reaching approximately 13% ± 0.5 for ATCC 19977 and 8% ± 1.2 for the clinical isolate (Figure 1e).

These results show that hMCs interact rapidly with both *Mab* morphotypes but only internalise a small proportion of bacteria under these in vitro conditions. Therefore, although these findings indicate limited internalization by hMCs, the potential contribution of adherent extracellular bacteria cannot be excluded.

### 3.2. M. abscessus Does Not Influence Mast Cell Viability or Degranulation

As hMCs participate in anti-mycobacterial activity by releasing mediators such as proteases and pro-/anti-inflammatory cytokines in sites of mycobacterial infection [14,15,18], understanding the effect of *Mab* exposure on hMC fitness and function becomes paramount. Given that hMCs interact with both *Mab* morphotypes but show limited bacterial internalisation, we next investigated whether *Mab* engagement with the MC surface affected cell viability or activation in vitro.

hMCs were exposed to both *Mab* strains at MOIs of 1, 5, 10, and 20 for 2 or 24 h and cell viability was analysed by flow cytometry (Supplementary Figure S1).

**Figure 2:**
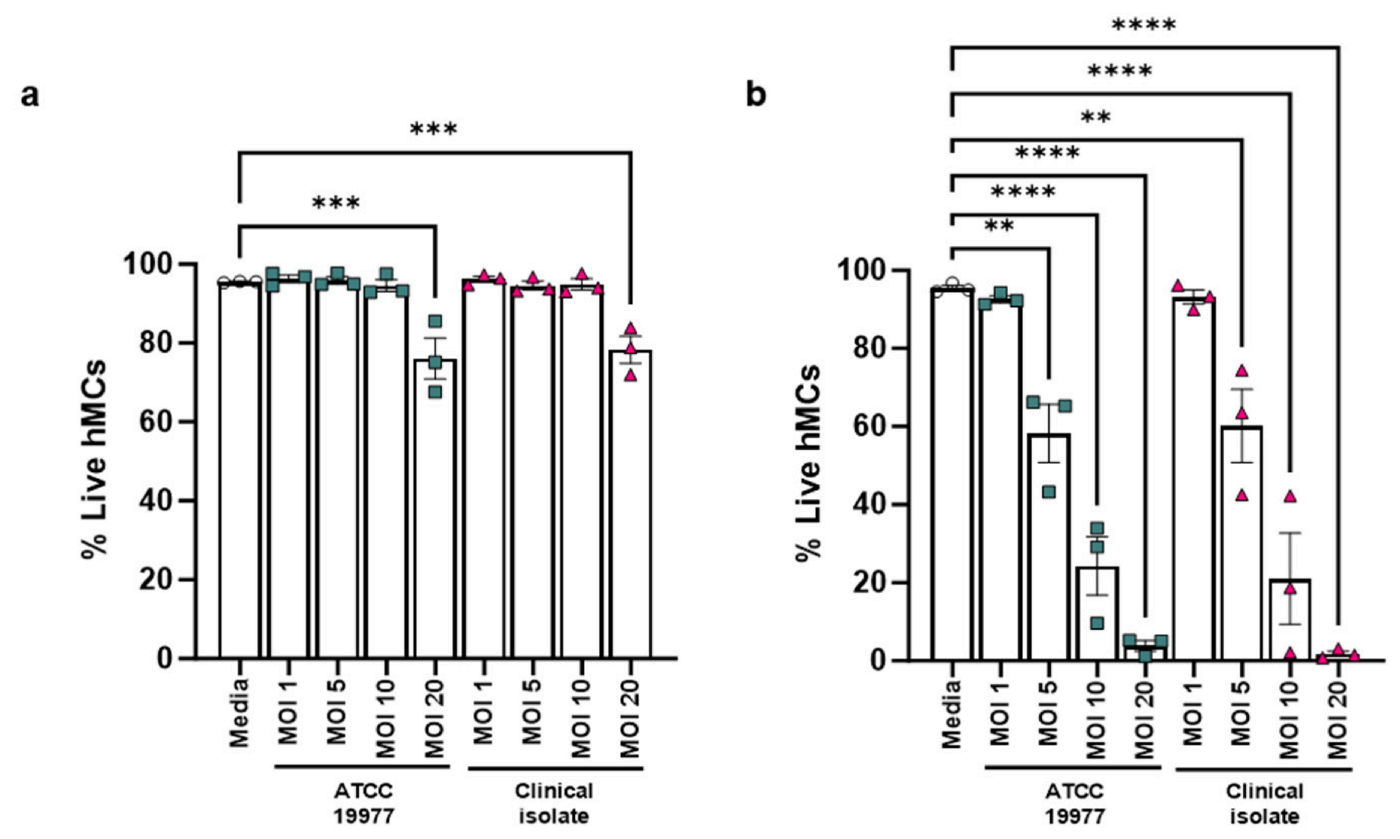
*Mab* affects the viability of mast cells in a time and dose-dependent manner. hMCs (0.5 × 10^6^ cells/mL) were stimulated for (**a**) 2 and (**b**) 24 h with *Mab* ATCC 19977 or the clinical isolate (MOI of 1, 5, 10, and 20). After stimulation, cells were stained with live/dead dye and analysed by flow cytometry. Graphs show the percentage of live hMC. Data are from at least three independent experiments and are expressed as means ± SEM (*n* = 3). Statistical analysis was performed using One-way ANOVA with Tukey’s multiple comparisons test. A *p* < 0.05 was considered statistically significant (**** *p* < 0.0001, *** *p* < 0.001, ** *p* < 0.01). White dots represent media, green squares represent the ATCC 19977 strain, and pink triangles represent the clinical isolate.

After 2 h of incubation, exposure to *Mab* did not significantly reduce MC viability at an MOI of 1, 5, and 10. At an MOI of 20, both strains caused a ~25% reduction in the number of viable cells (Figure 2a). In contrast, prolonged incubation for 24 h markedly affected cell viability for all MOIs, except MOI of 1, decreasing viability by approximately 40, 80, and 95% at MOIs of 5, 10, and 20, respectively (Figure 2b).

These results indicate that short-term exposure to *Mab* does not substantially affect MC viability, whereas prolonged exposure reduces cell survival in an MOI-dependent manner.

**Figure 3:**
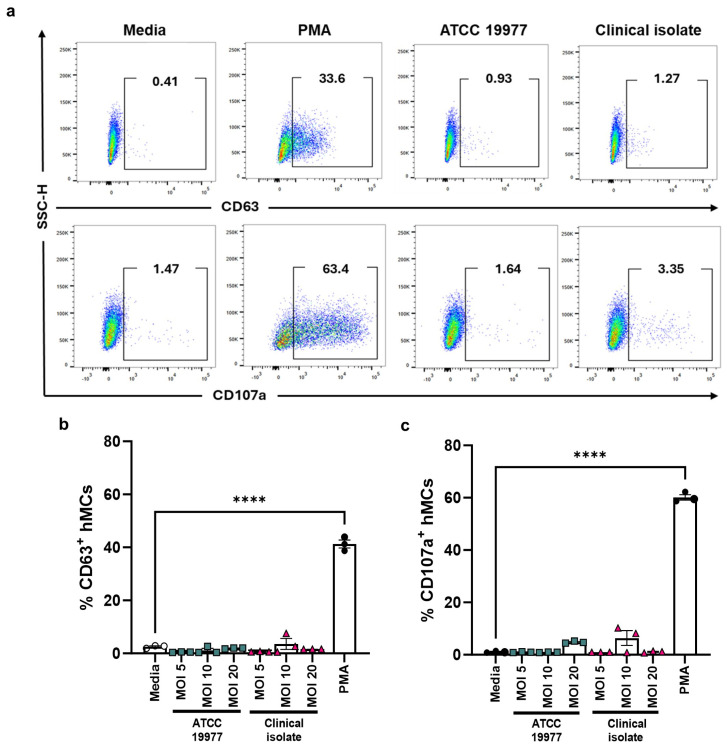
Exposure to *Mab* does not promote degranulation of hMCs. hMCs were stimulated for 2 h with *Mab* ATCC 19977 or the clinical isolate (MOI of 5, 10, and 20). After stimulation, cells were stained with Live/Dead dye (live cell gating), CD63 and CD107a antibodies (assessing degranulation), and analysed by flow cytometry. (**a**) An example of a dot plot showing hMCs, CD63, and CD107a-positive cells after incubation with *Mab*. Graphs show the percentage of (**b**) CD63, and (**c**) CD107a positive cells after bacterial stimulation. Data are from three independent experiments and are expressed as means ± SEM, (*n* = 3). Statistical analysis was performed using One-way ANOVA with Tukey’s multiple comparisons test. A *p* < 0.05 was considered statistically significant (**** *p* < 0.0001). White and black dots represent media and PMA controls, respectively; green squares represent the ATCC 19977 strain, and pink triangles represent the clinical isolate.

We next assessed whether *Mab* induced MC activation by measuring degranulation. hMCs were exposed to ATCC 19977 or the clinical isolate at MOI of 1, 5, 10, and 20. After 2 h of stimulation, membrane expression of CD63 and CD107a, used as markers of MC activation, was measured by flow cytometry (Figure 3a).

Exposure to *Mab* strains did not increase the proportion of MCs expressing CD63 or CD107a (Figure 3b,c), indicating that *Mab* does not induce MC degranulation in vitro.

In conclusion, hMCs do not seem to degranulate or mount extracellular bacterial killing responses to *Mab* exposure in vitro, irrespective of the strain tested.

### 3.3. M. abscessus Primarily Induces ROS Production, with Modest Increases in Granzyme B and IL-1β Expression in Human Mast Cells

Alternative methods for mycobacterial clearance include reactive oxygen species production, which MCs are known to produce following exposure to *Mtb* and BCG [16,28]. To assess the role of hMCs in pathogen elimination through oxidative stress, we measured ROS levels following exposure to *Mab*.

Cells were pre-loaded with the fluorescent probe for intracellular ROS detection, DHR, and exposed to either the *Mab* ATCC 19977 strain or the clinical isolate at MOIs of 1, 5, 10, and 20 for 2 h, then analysed by flow cytometry.

**Figure 4:**
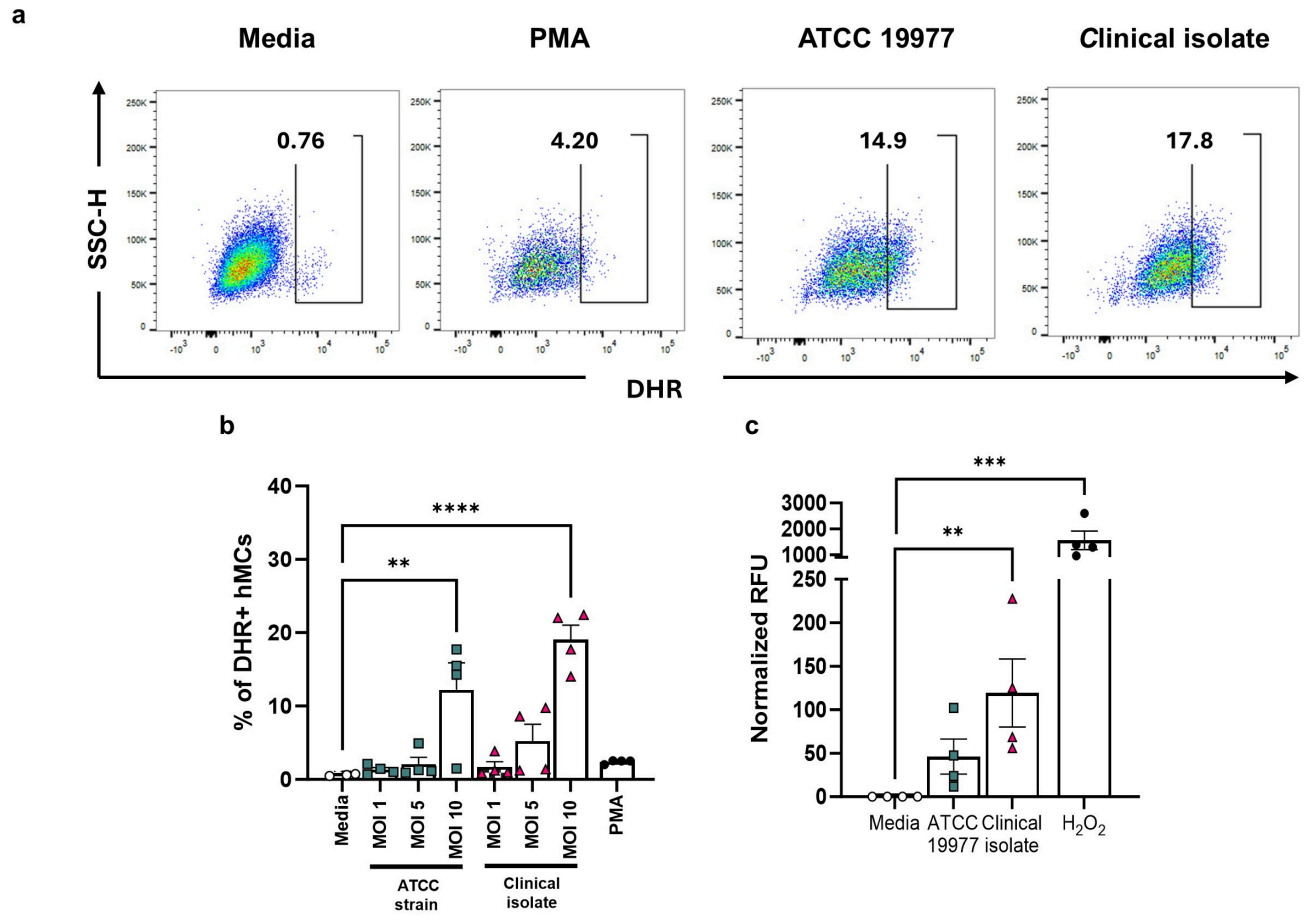
hMC ROS production upon exposure to *Mab* is strain and MOI-dependent. hMCs (0.5 × 10^6^ cells/mL) were pre-loaded with 5 µM DHR dye and then stimulated for 2 h with *Mab* ATCC 19977 or the clinical isolate (MOI of 1, 5, 10, and 20). After stimulation, hMCs were stained with live/dead dye. (**a**) An example of a dot plot showing hMCs after incubation with *Mab* at an MOI of 10. The graph shows the percentage of (**b**) DHR-positive cells after bacterial stimulation. Fluorescence intensity was measured using an Infinite 200 PRO plate reader (em:530/ex:488). (**c**) The graph shows the normalised fluorescence intensity at 2 h post-exposure. Data are from four independent experiments and are expressed as means ± SEM (*n* = 4). Statistical analysis was performed using One-way ANOVA with Tukey’s multiple comparisons test. A *p* < 0.05 was considered statistically significant (**** *p* < 0.0001, *** *p* < 0.001, ** *p* < 0.01). White and black dots represent media and PMA/H_2_O_2_ controls, respectively; green squares represent the ATCC 19977 strain, and pink triangles represent the clinical isolate.

As shown in Figure 4a, interaction between *Mab* and hMCs increased the proportion of DHR+ cells across all MOIs. For ATCC 19977 at an MOI of 10, values ranged from 1.5% to 17% DHR+ cells, while the clinical isolate produced values from 14% to 22% (*p* = 0.0023 and *p* < 0.0001, respectively). The percentage of DHR+ cells increased with higher MOI. Although exposure to the clinical isolate resulted in a higher percentage of DHR+ cells compared to the ATCC 19977 strain, this difference was not statistically significant (Figure 4b). While PMA stimulation is known to trigger ROS production in macrophages [29], hMCs did not exhibit an increase in DHR+ cells upon PMA stimulation compared to the media control.

An alternative technique was employed to validate the observed increase in ROS production. hMCs were incubated under similar conditions, and the fluorescence intensity of the entire well, including both cells and supernatant, was measured using a plate reader.

Consistent with the flow cytometry results in Figure 4b, we observed a significant increase in fluorescence intensity in cells cultured with the clinical isolate (Figure 4c). In contrast, the ATCC 19977 strain did not induce a significant increase in ROS.

These findings demonstrate that ROS production in hMCs upon *Mab* exposure is strain-dependent, with the clinical isolate eliciting the highest response.

To identify other immunological activities triggered by *Mab* exposure, we investigated whether the expression of cytokines or other intracellular mediators was affected in hMCs. To this purpose, we incubated cells for 12 h with the *Mab* clinical isolate at an MOI of 10. The intracellular levels of IL-1β, Granzyme B, IL-13, GM-CSF, CXCL10, TNF-α, IFN-γ, and IL-8 were then quantified by flow cytometry (Supplementary Figure S3).

**Figure 5:**
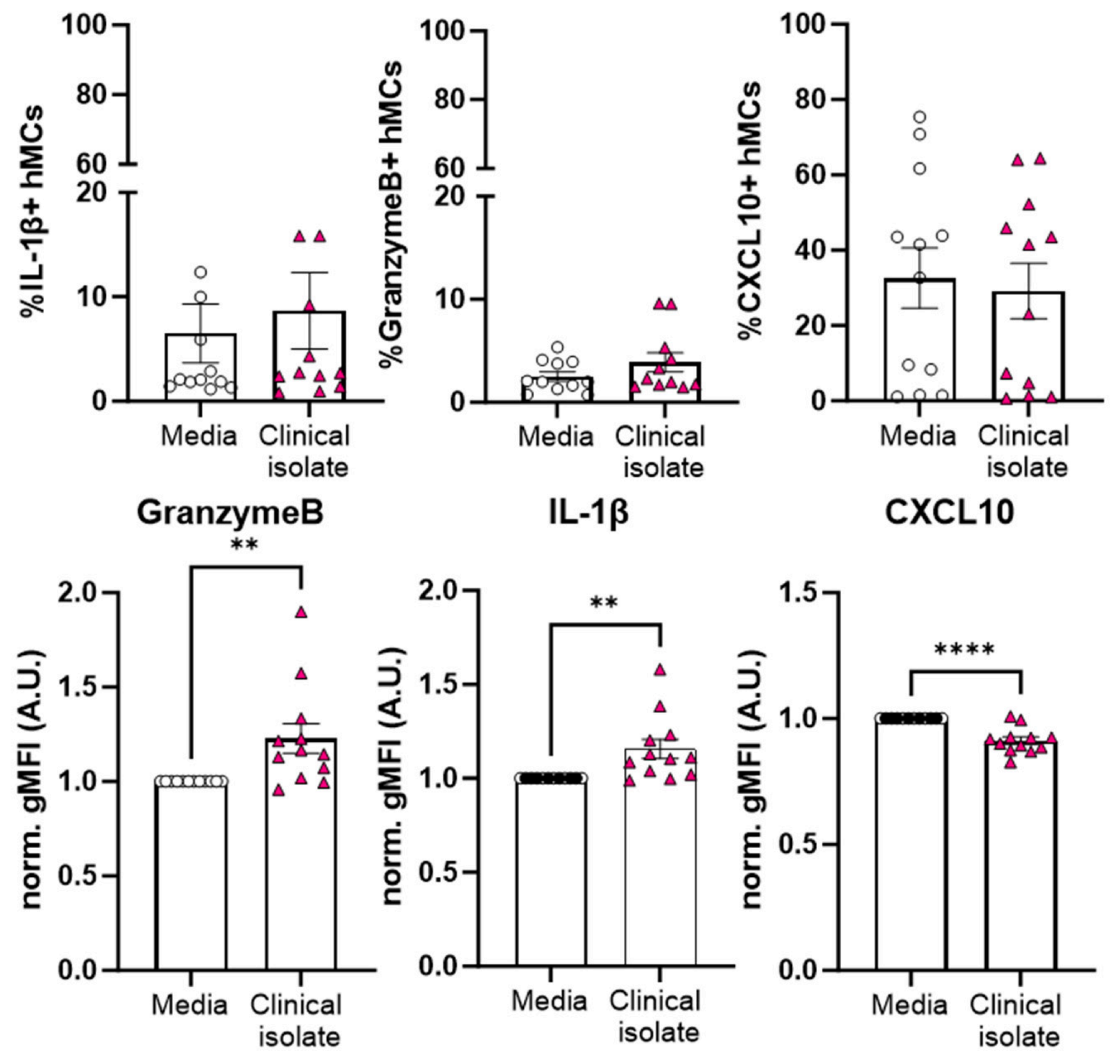
Intracellular mediator expression in hMCs after exposure to *Mab*. hMCs were stimulated for 12 h with *Mab* clinical isolate (MOI:10). After stimulation, cells were stained intracellularly with anti-CXCL10, IL-1β, and Granzyme B antibodies, and their expression was analysed by flow cytometry. Graphs show the geometric mean fluorescence intensity (normalised to the media control) of the different cytokines after bacterial stimulation. Data are from twelve independent experiments and expressed as means ± SEM (*n* = 12). Statistical analysis was performed using the Mann–Whitney test (normalised gMFI). A *p* < 0.05 was considered statistically significant (**** *p* < 0.0001, ** *p* < 0.01). White dots represent media control while pink triangles represent the clinical isolate.

Exposure to the *Mab* clinical isolate did not increase the frequency of cytokine-positive cells for any mediator. However, analysis of geometric mean fluorescence intensity (gMFI; Figure 5) indicated a modest increase in the intracellular Granzyme B and IL-1β expression levels following *Mab* exposure. In contrast, CXCL10, GM-CSF, IFN-γ, and IL-13 exhibited a slight reduction in expression.

Overall, these findings indicate that hMCs contribute to *Mab* immunity primarily via ROS production. Although a modest increase in intracellular responses related to Granzyme B and IL-1β was detected, the functional significance of these changes remains to be elucidated.

### 3.4. CD18 and CD11c Receptor Expression Changes over Time upon M. abscessus Exposure

*Mab* can interact with several cellular receptors, promoting surface adhesion and intracellular uptake [30]. However, to date, the specific receptors through which *Mab* interacts with MCs have not yet been elucidated. To explore potential receptors involved in hMC-*Mab* interactions, we investigated the expression levels of selected membrane receptors known to participate in MC activation or microbial recognition in mycobacterial/non-mycobacterial infections [12,30,31,32,33].

Following 2 h of *Mab* exposure, several receptors, including CD203c, MRGPRX2, Siglec-5/14, Siglec-6, Siglec-3 (CD33), DAP-12, CD53, L1CAM, ICAM-1, CD18, CD11c, CD11b, CD46, TLR2, and CD48, showed no significant modulation in hMCs (Supplementary Figure S4).

**Figure 6:**
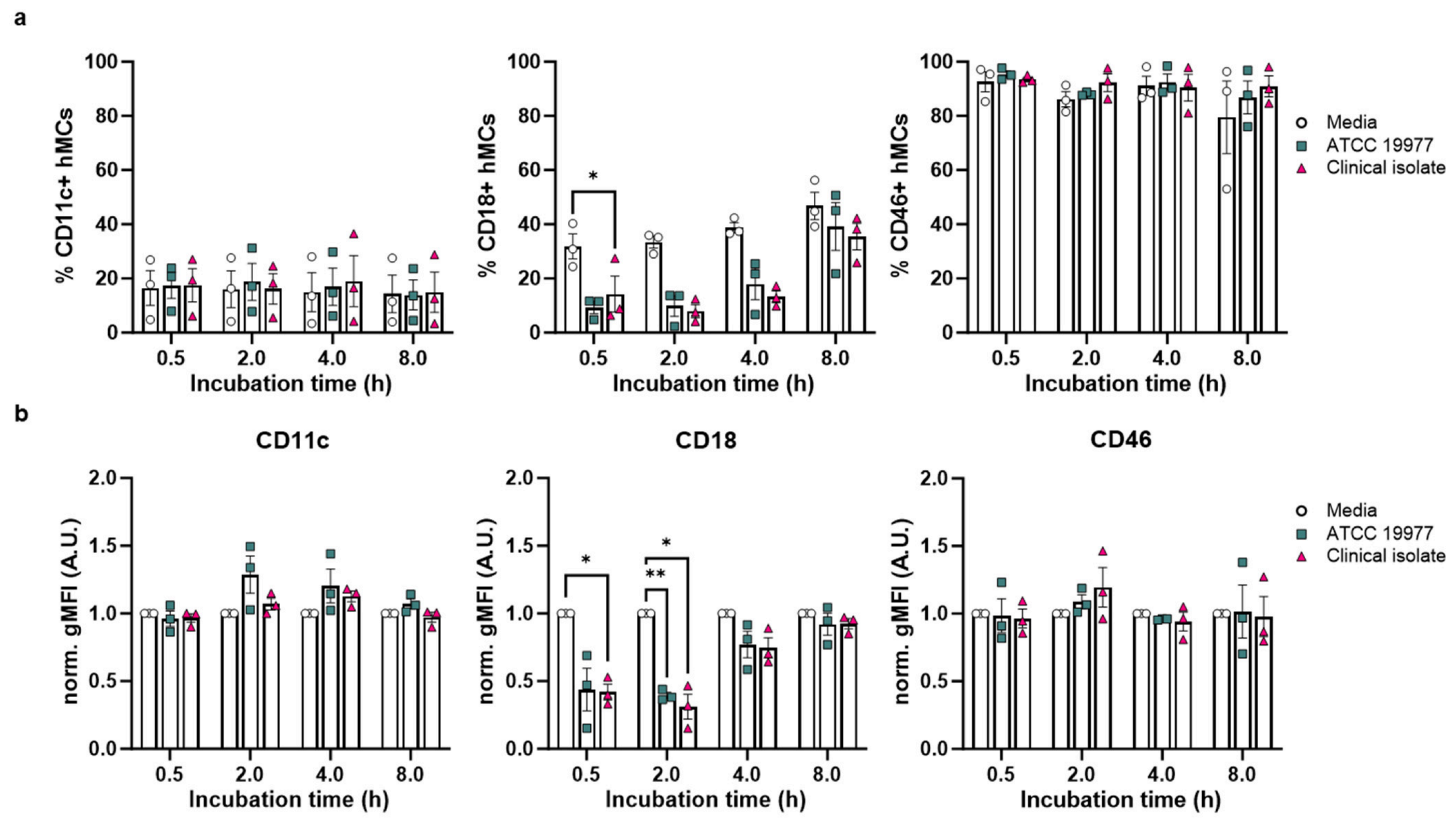
Modulation of CD18, CD11c and CD46 surface expression in hMCs after exposure to *Mab*. hMCs were stimulated for up to 8 h with *Mab* ATCC 19977 or the clinical strain (MOI of 10). After stimulation, cells were stained with anti-CD46, CD11c and CD18 antibodies, and their surface expression was analysed by flow cytometry. Graphs show (**a**) the percentage of positive cells (**b**) the geometric mean fluorescence intensity (normalised by the non-primed media control) at 0.5, 2, 4, and 8 h, and for CD18, CD11c and CD46 at 0.5, 2, 4, and 8 h of bacterial stimulation. Data are from three independent experiments and expressed as means ± SEM, *n* = 3. Statistical analysis was performed using one-way ANOVA with Tukey’s multiple comparisons test (% of positive cells) or Friedman test with Dunn’s multiple comparisons test (gMFI). A *p* < 0.05 was considered statistically significant, ** *p* < 0.01, * *p* < 0.05).

As no receptor modulation was observed after 2 h of *Mab* exposure, the incubation period was extended to 8 h to determine whether earlier analyses had missed delayed changes in surface expression. Complement receptors CR3 (CD11b/CD18) and CR4 (CD11c/CD18) were selected for further analysis due to their reported involvement in MC responses to other pathogens [34,35,36]. hMCs exposed to *Mab* showed a rapid decrease in the percentage of CD18+ cells within 30 min, particularly in response to the clinical isolate. This reduction was transient, as no significant differences were detected at 2 or 4 h post-infection, and CD18 expression returned to baseline levels by 8 h. In contrast, the percentages of CD11c+ and CD46+ cells did not show significant changes (Figure 6a).

Analysis of receptor expression levels by gMFI showed a slight decrease in CD46 expression after 4 h of *Mab* exposure, which recovered by 8 h (Figure 6b). CD11c expression increased at 4 h and returned to baseline by 8 h, while CD18 expression also decreased at 4 h and subsequently recovered.

These results indicate that *Mab* induces transient and time-dependent modulation of selected MC surface receptors, particularly CD18, suggesting dynamic receptor regulation during the early stages of bacterial interaction.

Recent studies have shown that an IL-33-enriched microenvironment enhances the expression of pathogen-recognition receptors in hMCs, including CD48 and TLR2, facilitating interactions with other mycobacteria such as BCG [24]. Based on this, we investigated whether cytokine priming with IL-33 and IFN-γ, cytokines mimicking alarmin-rich and pro-inflammatory environments as observed during active *Mab* disease or in the presence of comorbidities like cystic fibrosis [37,38,39,40], could enhance the expression of receptors potentially involved in *Mab* recognition by hMCs.

Transcriptomics data of 24-h-primed hMCs with IL-33 or IFN-γ in the absence of *Mab* suggests cytokine-specific changes in transcription of hMC genes linked to bacteria uptake and intracellular killing. We observed upregulation of FPR1, NOD2 and TLR2 by both IL-33 and IFN-γ pre-incubation. Conversely, increased transcription of CD48 and TLR4 was observed only after IFN-γ priming, while CD209, CLEC7A, FPR2 and MRC1 were uniquely upregulated in response to IL-33 priming. Conversely, receptors like CD46 and ITGB2/CD18 were either downregulated in response to IL-33 or not significantly changed compared to unstimulated controls (Supplementary Figure S5a). This demonstrates that the cytokine milieu can produce significant changes in hMCs response, predominantly in innate pattern recognition receptors and the phagocytic uptake machinery.

We sought to further validate said observations by direct measuring of receptor expression levels of particular interest for *Mab* surface recognition [30] in flow cytometry, namely CD18, CD46, CD48, TLR2 and TLR4. Priming with IFN-γ increased the gMFI of CD46 and TLR2 expression on hMCs, IL-33 increased CD48 expression, whereas CD18 expression remained unchanged (Supplementary Figure S5b).

In conclusion, cytokine priming modulates the expression of selective receptors relevant in hMC mycobacterial recognition, specifically TLR2/4, CD46 and CD48, while not affecting significantly CD18 expression.

### 3.5. Human Mast Cell Interaction with M. abscessus Involves CD18 and CD46 Receptors

As our microscopy work demonstrated direct physical interactions between MCs and both *Mab* strains, and *Mab* exposure induced surface expression modulation of some receptors, namely CD18 and CD46, we next assessed the contribution of these receptors to hMC-*Mab* interactions by using selective receptor blocking.

hMCs were incubated with blocking antibodies against CD18, CD46, or CD48 for 1 h before challenge with CFSE-labelled *Mab* clinical isolate (MOI of 10), and live-cell imaging was performed using fluorescence microscopy to measure the number of contacts between *Mab* and hMC membrane or membrane protrusions (representative videos of each condition are included as Supplementary Videos S3–S6). As CD18 expression in MCs was heterogeneous among donors (Supplementary Figure S4), both CD18-positive and CD18-negative hMC cultures were included in the analysis.

**Figure 7:**
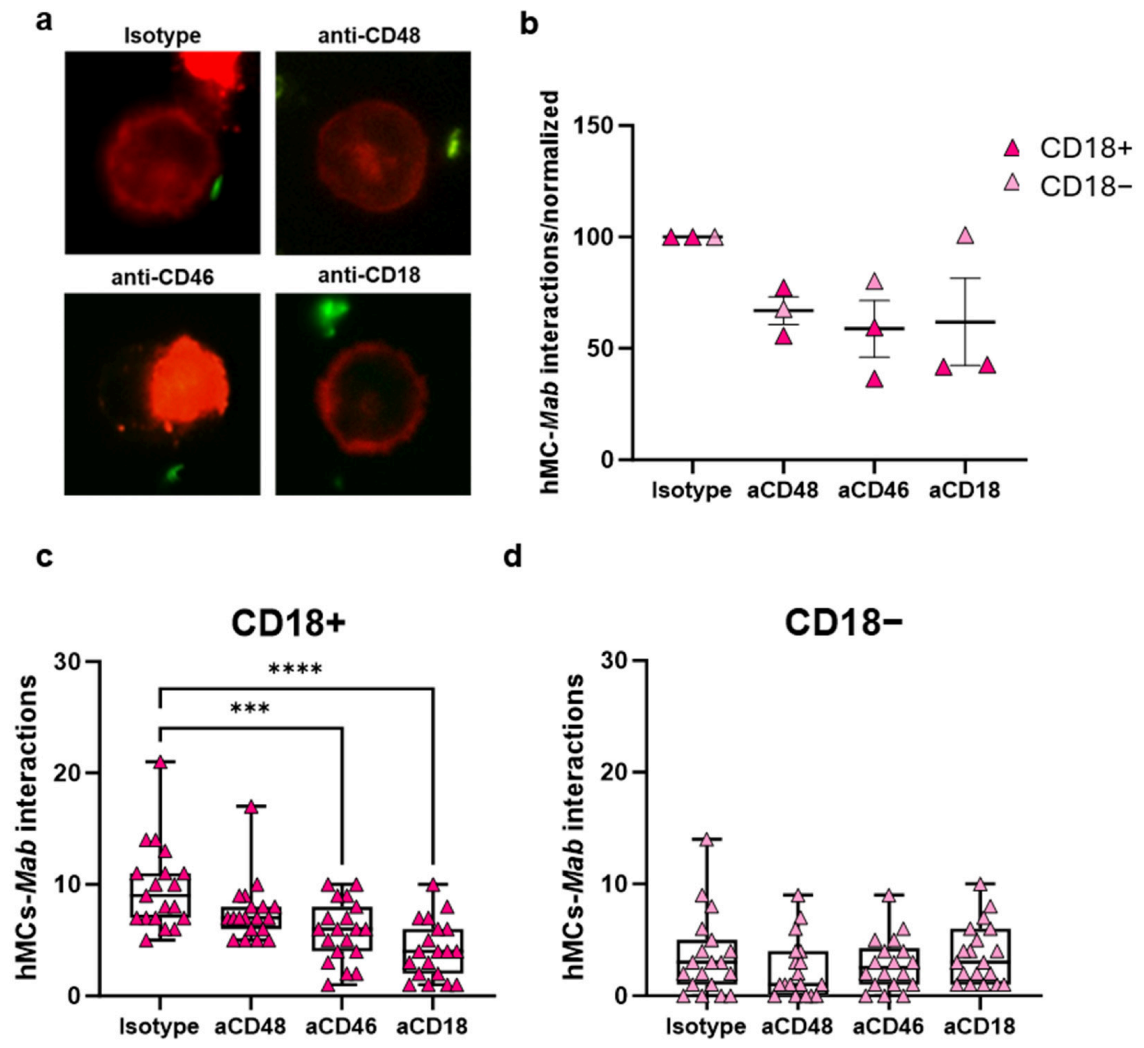
CD18 and CD46 mediate interactions between *Mab* and hMCs. hMCs were incubated for 1 h with anti-CD48, anti-CD46, anti-CD18, and isotype antibodies. Live-cell imaging was performed as previously described. (**a**) Representative images of hMC-*Mab* interactions following incubation with the different antibodies. (**b**) The number of interactions was manually counted and normalised relative to the isotype control for each experiment. Representative graph showing the number of interactions in (**c**) CD18+ (dark pink triangles) and (**d**) CD18-cells (light pink triangles) treated with each blocking antibody. Data shown represent individual cells from one representative experiment and are presented for descriptive purposes. Data were analysed using FIJI, and single contacts between bacilli and the MC membrane or cellular protrusions were considered an interaction. A total of *n* = 19 cells per power field were analysed in each condition. Statistical analysis was performed using one-way ANOVA with Tukey’s multiple comparisons test. A *p* < 0.05 was considered statistically significant (**** *p* < 0.0001, *** *p* < 0.001).

The *Mab* clinical isolate established direct contacts with the hMC membrane under all treatment conditions (Figure 7a). Treatment with blocking antibodies against chosen receptors reduced bacterial contacts, however, differences between isotype and blocking antibodies in the overall tested subjects were not statistically significant (Figure 7b).

In CD18+ donors, hMC-*Mab* contacts were approximately 10 interactions per field in the isotype control group (Figure 7c), while CD18-MCs showed fewer baseline interactions, not further reduced by antibody treatment (Figure 7d).

In CD18+ MCs, blockade of CD18 and CD46 reduced bacterial interactions by approximately 60% and 50%, respectively (Figure 7c). In CD18-MCs, inhibition of hMC-*Mab* interactions following CD48 blockade was ~30%, CD46 blockade produced a smaller ~20% reduction, although not significant, while CD18 blockade had no effect on CD18-cells, as expected (Figure 7d).

These results indicate that CD18 and CD46 contribute to *Mab* recognition by hMCs and mediate a substantial proportion of early cell-bacteria interactions. In the absence of CD18, other receptors, including CD48, may partially compensate for bacterial recognition.

### 3.6. The Lack of CD18 Expression in Mast Cells Is Associated with Impaired ROS Production in Response to M. abscessus

To further assess the contribution of CD18 to ROS production during *Mab* exposure, CD18-positive and deficient hMCs were pre-loaded with DHR and infected with *Mab* at MOI of 10 and analysed by flow cytometry.

**Figure 8:**
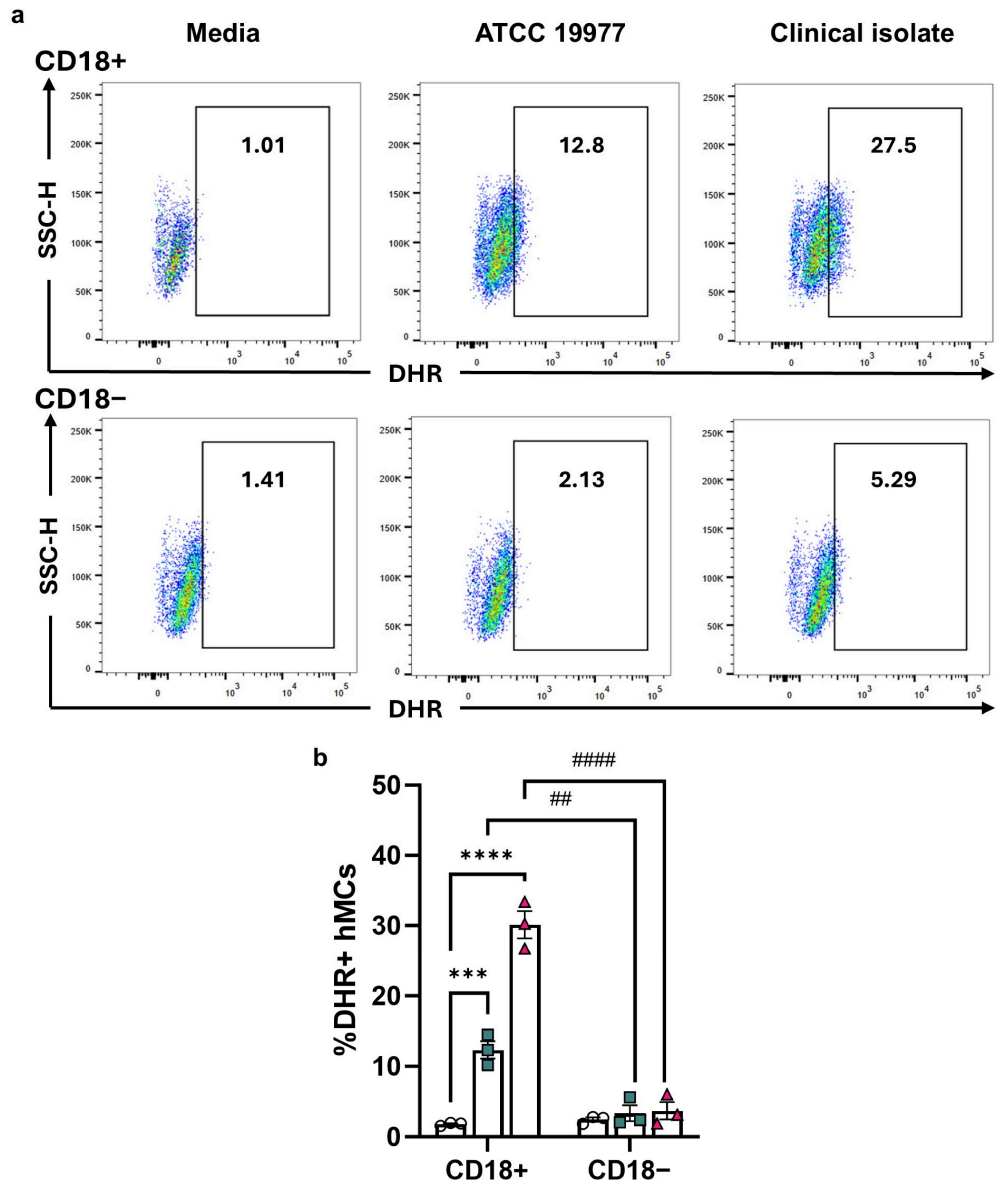
CD18 mediates ROS production upon exposure to *Mab*. CD18-expressing (CD18+) and CD18-deficient (CD18-) hMCs were pre-loaded with 5 µM DHR dye and then stimulated for 2 h with *Mab* ATCC 19977 or the clinical isolate (MOI of 10). After stimulation, ROS production was quantified as the percentage of DHR+ cells by flow cytometry. (**a**) An example of a dot plot showing hMCs (CD18+ and CD18-) after incubation with media or *Mab* at an MOI of 10. (**b**) The graph shows the percentage of DHR-positive cells after bacterial stimulation. Data show the mean of three independent experiments, ± SEM. Statistical analysis was performed using two-way ANOVA with Tukey’s multiple comparisons test. * indicates differences between treatment groups; ^#^ indicates differences between MC groups under the same treatment (**** *p* < 0.0001, *** *p* < 0.001, ^####^ *p* < 0.0001, ^##^ *p* < 0.01). White dots represent media, green squares represent the ATCC 19977 strain, and pink triangles represent the clinical isolate.

When analysing CD18-expressing hMCs, exposure to *Mab* increased the proportion of DHR+ cells (Figure 8a). hMC stimulation with the ATCC 19977 strain increased the percentage of DHR+ cells from 1.8% ± 0.1 to 12.3% ± 1.2 (*p* < 0.001), whereas their exposure to the clinical isolate produced a stronger response, increasing the percentage of DHR+ cells to 30.1% ± 2.0 (*p* < 0.0001) (Figure 8b).

In contrast, CD18-deficient MCs showed a markedly reduced ROS response to all *Mab* strains. This reduction was significant across all conditions and was most pronounced following exposure to the clinical isolate (89.62% reduction compared to CD18-expressing hMCs, *p* < 0.0001).

As cytokine priming increases the expression of receptors such as TLR2, CD46, and CD48, which can participate in ROS generation, we also assessed cytokine-driven boosting and/or inhibition of ROS production in both CD18+ and CD18-donors. As expected, priming with IL-33 or IFN-γ did not significantly increase the proportion of DHR+ cells in response to any *Mab* strain compared to non-primed cells (Supplementary Figure S6).

Collectively, these results further suggest that CD18 expression contributes substantially to ROS production in hMCs following *Mab* exposure, as its absence was associated with significantly reduced ROS levels across all strains tested.

## 4. Discussion

This study shows that MCs mount a rapid, CD18- and CD46-receptor-dependent intracellular response to both smooth and rough *Mab* morphotypes. *Mab* presence stimulates intracellular ROS generation, Granzyme B, and IL-1β production in the MCs. To our knowledge, this study is the first to investigate the role of hMCs in the immune response to *Mab*.

MC cultures were generated from circulating pre-committed CD117+ MC progenitors, which are substantially different from hMC cell lines or animal-derived MCs [41]. Despite its reductionism, this model has allowed us to replicate both in vitro physiological responses to *Mab* and capture donor-to-donor variability in response, which is not possible with cell lines or murine models [41,42]. However, due to limited evidence on MCs’ role in immune control of *Mab* infection, our findings only partially align with studies conducted on other MC models and mycobacteria.

In our experimental model, short-term exposure to *Mab* did not affect cellular viability. However, prolonged culture caused significant hMC death at concentrations ≥ MOI of 20, irrespective of the morphotype. This likely results from an immune evasion mechanism used by *Mab*, also shown in macrophages [43]. This mechanism seems to be relevant only for the rough morphotype due to the lack of GPLs, which promote cellular apoptosis, autophagy, necrosis, and pyroptosis. We speculate that this discrepancy arises from the activation of a distinct cellular death pathway in hMCs, independent of GPL composition or cell-specific differences in responses to different *Mab* morphotypes, worthy of further exploration.

When using RBL-2H3/HMC-1 cell lines and animal models, MCs seem to play a bigger role in extracellular bacterial clearance through degranulation and TNF production in response to *Mtb*, BCG, *M. marinum,* and *M. smegmatis* [13,16,17,44] compared to what we observed using hMCs. This suggests not only differences in mycobacterial species, but also model-specific differences in degranulation induction. Our results align more closely to experimental BCG infection models using hMCs, suggesting minimal phagocytic activity, shorter duration of hMC-*Mab* interaction, and limited degranulation potential [24].

Classically, MCs contribute to pathogen clearance through phagocytosis-mediated intracellular killing and degranulation-dependent release of antimicrobial mediators [36,45,46]. The absence of these antibacterial activities in response to Mab may also reflect a lower virulence of this pathogen compared to that of tuberculous mycobacteria [47,48,49], as *Mab* predominantly causes disease in immunocompromised individuals and patients with underlying structural lung disease [2].

Interestingly, hMCs display increased ROS production following *Mab* exposure, which facilitates both extracellular and intracellular bacterial clearance, particularly evident with the rough morphotype. This could be due to changes in the amount of GPLs in the cell wall, which could facilitate contact and engagement of surface receptors and the mounting of protective responses, despite the limited phagocytic potential [50,51].

The role of ROS in host defence against mycobacterial infections has been widely documented [43,52,53]. Increased oxidative stress can promote macrophage apoptosis, contributing to bacterial control and limiting pathogen dissemination [43]. However, excessive ROS production may also induce necrotic cell death and tissue damage [54], a hallmark of skin lesions associated with *Mab* infection [55].

Furthermore, hMCs display increased intracellular expression of IL-1β and Granzyme B upon *Mab* exposure, suggesting activation of pro-inflammatory antimicrobial responses. IL-1β plays a fundamental role in barrier immunity, as it is linked to protective immune responses against mycobacteria, particularly important in controlling early stages of infection [56,57]. In mycobacterial lesions, increased Granzyme B expression by neutrophils, a serine protease shared by phagocytic immune cells and hMCs, is associated with higher bacterial burden [58] and more favourable granuloma outcomes [59]. This protease is also linked to pyroptosis [60], which could explain the reduction in hMC viability observed with prolonged *Mab* exposure [61] and, in part, the downward trend in the release of mediators like CXCL10, GM-CSF, IFN-γ, and IL-13.

While the expression levels of most investigated hMC surface receptors did not change because of *Mab* exposure, a slight reduction in CD18 expression was observed. Recent evidence suggests a prominent role of complement receptors CR3/CR4 in *Mab* uptake, as complement receptor subunits CD18, CD11b (CR3), and CD11c (CR4) were involved in bacterial recognition and internalisation in human macrophages [62] and neutrophils [63]. The low-level expression of CD11c by hMCs further supports the hypothesis that bacterial internalisation cannot occur effectively, unlike in macrophages, but partial *Mab* recognition could still occur through CD18.

The early reduction in CD18 expression observed within 30 min from initial *Mab* inoculation matches earlier findings of rapid interaction between hMCs and *Mab* and further hints at rapid surface regulation mechanisms. As CD18 in leukocytes can undergo proteolytic cleavage by cysteine proteases, which can lead to the loss of the extracellular domain [64], we speculate that hMCs could also operate in a similar manner, as cysteine proteases like cathepsin B are usually upregulated in pro-inflammatory conditions such as psoriasis [65].

Since hMCs are known to sense a plethora of surrounding cytokines which can significantly influence their function and phenotype [22], we have explored the effect of cytokine priming on both receptor transcription and expression modulation. Cytokine priming with IL-33 and IFN-γ only significantly affected CD46, CD48, and TLR2 transcription and expression, while CD18 was not significantly modulated, consistent with previous reports [24].

The study of the involvement of CD18, CD46, and CD48 receptors in hMCs-*Mab* interactions showed significant differences when using blocking antibodies only in CD18+ cultures, where the addition of anti-CD46 and anti-CD18 antibodies significantly reduced the number of hMC-*Mab* interactions compared to the isotype control. This hints at a crucial role of CD18 in mediating first contact with *Mab*, as interactions are almost abated in its absence. Similarly, CD46, an accessory complement regulatory protein, is also recognised as a broad pathogen-binding receptor involved in interactions with multiple microorganisms, including *Neisseria gonorrhoeae*, *Neisseria meningitidis*, and several viruses [66], showing significant downregulation upon infection [67].

Interestingly, CD46 seems to play a redundant role in increasing the likelihood of hMC-*Mab* interaction; however, this effect is significant only in CD18+ cultures. The synergistic effect of multiple receptors has been previously reported with Dectin-1 and TLR2 during *Mab* infection in macrophages [68], demonstrating physical interaction and co-localisation, suggesting the existence of a complex regulatory network involved in *Mab* recognition. Conversely, CD48 does not seem to play a major interactive role. This contrasts with evidence from other mycobacteria, particularly *Mtb* and BCG, where CD48 expression drives mycobacterial interactions with hMCs, particularly in the presence of IL-33 priming [24].

We then tested the functional impact of CD18 presence/absence in ROS production, demonstrating reduced ROS production in CD18-donors compared to CD18+ hMC cultures. This further solidifies our hypothesis that this peculiar integrin plays a major role in *Mab* recognition and clearance mechanisms mediated by hMCs. CD18 is widely expressed by several immune cells beyond MCs that participate actively in granuloma formation [69,70], including macrophages, neutrophils, and subsets of B, T, and NK cells [71]. Investigating the contribution of this integrin in the establishment of the immune response against *Mab* infection becomes paramount.

Although not demonstrated in hMCs or mycobacteria, evidence suggests CD18-dependent ROS production can be driven by homotypic ERK aggregation mediated in *Candida albicans* infection models [72]. Consistent with the lack of changes observed in surface receptor expression, cytokine priming with IL-33 and IFN-γ did not significantly increase ROS production in CD18+ cultures, while causing a significant increase in ROS production in IL-33-primed CD18- cultures, although of minimal magnitude. Based on current evidence, while in hMCs CD18 seems to play a major role in bacterial interaction and ROS production, the functional redundancy of CD18 has been reported in other NTM infections, where the complete absence of CD18 did not prevent *M. avium* uptake in macrophages [73], suggesting that receptors with similar intracellular pathways to CD18 could also participate in mycobacterial immune recognition.

While this study provides novel insights into MC responses to *Mab* infection, several limitations must be acknowledged. First, the experimental analyses were performed using anonymized donors with unknown gender and immune status. This lack of information may have introduced donor-to-donor variability, potentially affecting the observed responses. Second, the investigations focused exclusively on acute infection, as higher MOIs or extended co-incubation with *Mab* led to significant cytotoxicity, thereby restricting the applicability of findings to realistic infection scenarios.

Third, the experimental system did not include complement components, such as the opsonin iC3b, which binds *Mab* and facilitates CD18-mediated recognition and phagocytosis [74,75]. Incorporating complement in future studies may uncover additional MC effector functions, including enhanced degranulation, increased ROS production, or modified cytokine responses.

Additionally, the use of single bacterial strains per morphotype [76,77], and the lack of tissue validation limit the broader applicability of these findings, particularly given the frequent co-occurrence of polymicrobial infections in this context [78,79].

The clinical importance of understanding MC responses to *Mab* is particularly apparent in patients with cystic fibrosis, where *Mab* is a leading cause of chronic pulmonary infection [79] and presents significant therapeutic challenges [80]. Evidence indicates that the smooth morphotype initiates infection by colonizing the host and may transition to the rough morphotype as the disease progresses [4]. Both morphotypes can coexist and may evolve differently in response to host antimicrobial pressures [76]. In this study, only one strain representing each morphotype was analysed. Consequently, the observed differences between ATCC 19977 and the clinical isolate, including ROS production, cannot be solely attributed to morphotype and may also reflect strain-specific characteristics. Further research utilizing multiple strains or isogenic smooth/rough pairs is necessary to clarify the contribution of morphotype-associated factors to MC-mediated recognition and responses.

Future work should therefore prioritise elucidating the full scope of CD18-mediated effects, from intracellular signalling to responses in the presence of opsonised bacteria such as iC3b [74,75]. Co-culture and in vitro granuloma models [80,81] should be employed to better define MC contributions within the broader infection microenvironment, including interactions with macrophages, fibroblasts, and stromal cells [82]. Extending these findings to other NTMs will help clarify whether the recognition pathways identified here are unique to *Mab* or reflect broader mechanisms of MC-mediated antibacterial immunity. Furthermore, validation in relevant in vivo models is necessary to determine the physiological significance of CD18-mediated hMC responses during *Mab* infection. Should future mechanistic and in vivo studies corroborate these results, the findings could justify investigating CD18-targeting agents, including erlizumab and rovelizumab [83], as potential host-directed therapies for mycobacterial infections.

## 5. Conclusions

With this study, we have demonstrated that MCs respond to different *Mab* morphotypes through a complex, redundant receptor network involving CD18 and CD46. While in our model, we could not observe significant uptake and degranulation, *Mab* exposure induced ROS and increased intracellular Granzyme B and IL-1β production, particularly with the clinical isolate, indicating the modulation of inflammatory and oxidative pathways rather than direct bacterial killing. The findings indicate that MCs function as early innate immune sensors and may contribute to host defence against *Mab* at barrier sites, especially in the lung or skin, where *Mab* infections are most commonly established.

Further studies are needed to confirm these findings.

## Data Availability

The original contributions presented in the study are included in the article/Supplementary Materials. Further inquiries for the datasets generated for this study can be obtained from the corresponding author upon reasonable request.

## Supplementary Materials

The following supporting information can be downloaded at: https://www.mdpi.com/article/10.3390/cells15151385/s1, Figure S1: Assessment of blood progenitors-derived hMC: maturation and purity; Figure S2: Bacterial culture of two different strains of *Mab*: a reference bacterial strain (ATCC 19977) and a clinical isolate; Figure S3: Intracellular mediator expression in hMCs after exposure to *Mab*; Figure S4: Modulation of receptor expression in hMCs after exposure to *Mab*; Figure S5: IL-33 and IFN-ɣ promote the expression of pathogen-binding receptor genes on MCs; Figure S6: ROS production upon exposure to *Mab* is not affected by IL-33 or IFN-ɣ priming; Video S1: Representative live-cell imaging video showing WGA-labelled hMCs (red) exposed to CFSE-labelled *Mab* ATCC 19977 (green) at an MOI of 10; Video S2: Representative live-cell imaging video showing WGA-labelled hMCs (red) exposed to CFSE-labelled *Mab* clinical isolate (green) at an MOI of 10; Video S3: Representative live-cell imaging video showing WGA-labelled hMCs (red) pre-incubated for 1 h with anti-CD18 blocking antibody before challenge with CFSE-labelled *Mab* clinical isolate (green) (MOI:10); Video S4: Representative live-cell imaging video showing WGA-labelled hMCs (red) pre-incubated for 1 h with anti-CD46 blocking antibody before challenge with CFSE-labelled *Mab* clinical isolate (green) (MOI:10); Video S5: Representative live-cell imaging video showing WGA-labelled hMCs (red) pre-incubated for 1 h with anti-CD48 blocking antibody before challenge with CFSE-labelled *Mab* clinical isolate (green) (MOI:10); Video S6: Representative live-cell imaging video showing WGA-labelled hMCs (red) pre-incubated for 1 h with isotype antibody before challenge with CFSE-labelled *Mab* clinical isolate (green) (MOI:10).

## Abbreviations

The following abbreviations are used in this manuscript:

IL	Interleukin
*Mab*	*Mycobacterium abscessus*
MC	Mast cells
PMA	Phorbol 12-Myristate 13-Acetate
