# Supplementary material for: Mycobacterium abscessus Induces CD18-Dependent Reactive Oxygen Species Production in Human Mast Cells
Source: Cells. 2026 Jul 31;15(15):1385. doi: 10.3390/cells15151385 (PMC13465594; doi:10.3390/cells15151385)
Supplement: Supplementary file 1 [file cells-15-01385-s001.zip › suplemmentary material.pdf]

Supplementary Figures

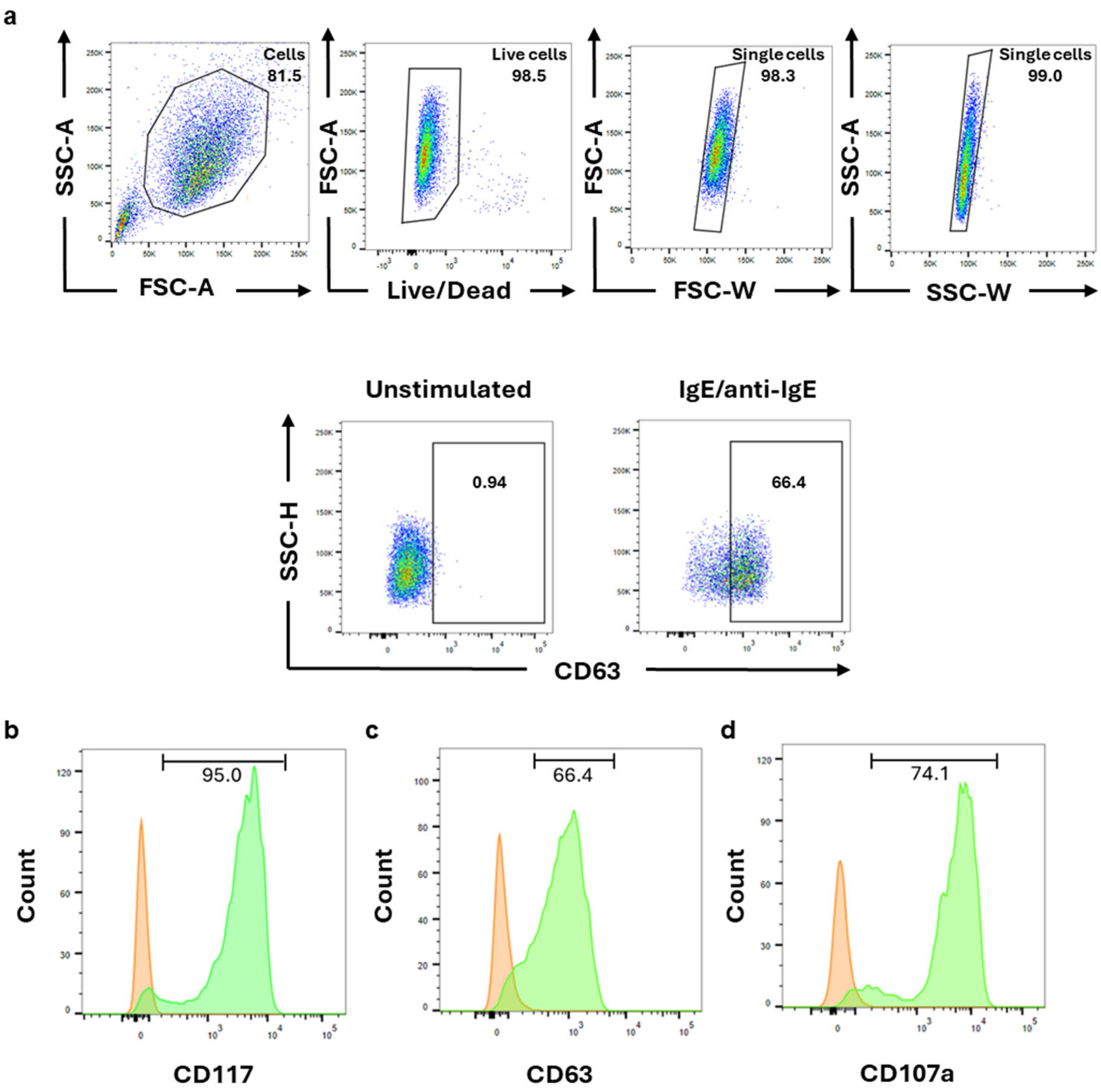

Figure S1. Assessment of blood progenitors-derived hMC: maturation and purity. CD117+ cells were isolated from PBMCs using magnetic-activated cell sorting (MACs) and then differentiated into MCs during 8 weeks of culturing in cell medium supplemented with different cytokines. Determination of maturation was assessed by flow cytometry. hMCs were sensitized overnight with IgE (1 µg/mL) and then activated for 30 min with anti-IgE (1 µg/mL) antibodies. (**a**) An overview of the gating strategy for the flow cytometry staining. hMCs were selected by the side scatter (SSC-A) vs forward scatter (FSC-A). Viable cells were determined by live/dead blue staining. Single cells were gated using FSC-A versus FSC-H and SSC-A vs SSC-W. Degranulation was determined by quantifying the expression of the surface markers CD63 and LAMP-1 (CD107a) by flow cytometry. The expression of specific MC surface marker CD117 was also determined. Each marker was gated based on Fluorescence minus one (FMO) staining controls. Histograms show (**b**) CD117, (**c**) CD63 and (**d**) CD107a (green) positive cell counts compared to unstained controls (orange).

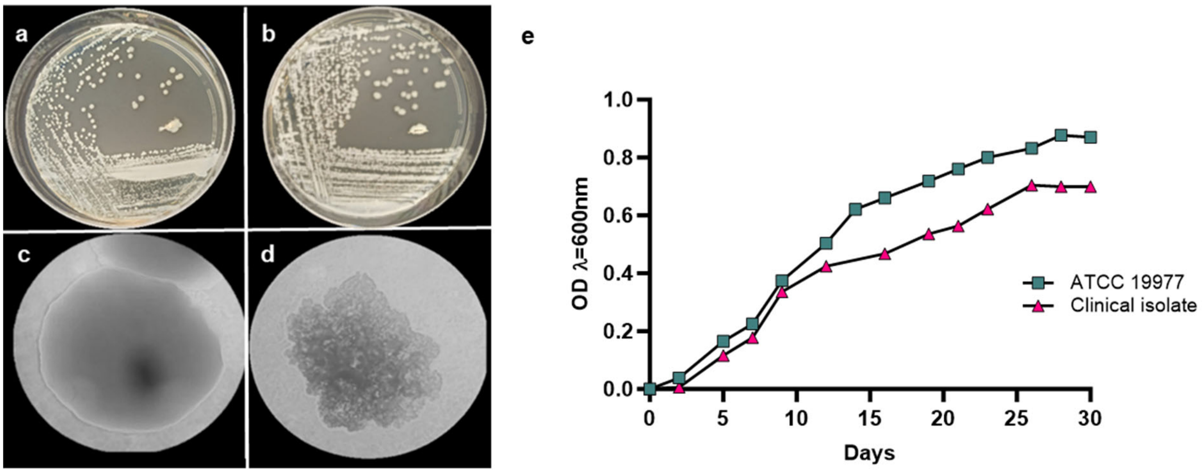

Figure S2. Bacterial culture of two different strains of *Mab*: a reference bacterial strain (ATCC 19977) and a clinical isolate. (**a-d**) Representative images of *Mab* colonies growing in solid medium, Middlebrook 7H11, after 4 days of incubation at 37 °C. (**a**) *Mab* ATCC 19977 strain grows in circular, bright, and white-pigmented small colonies. (**b**) the clinical strain colonies are bigger, dry, rough, and white-pigmented. Light microscope micrograph (10x magnification) of one bacterial colony. (**c**) ATCC 19977 presents as round with a well-defined border, whereas (**d**) the clinical isolate has an undefined shape with a corrugated border. (**e)** Growth curves of ATCC 19977 and the clinical strain were obtained by measuring the optical density every two days after inoculation of one colony in the medium Middlebrook 7H9, following incubation at 37 °C for 30 days. The data are representative of n=2 independent experiments.

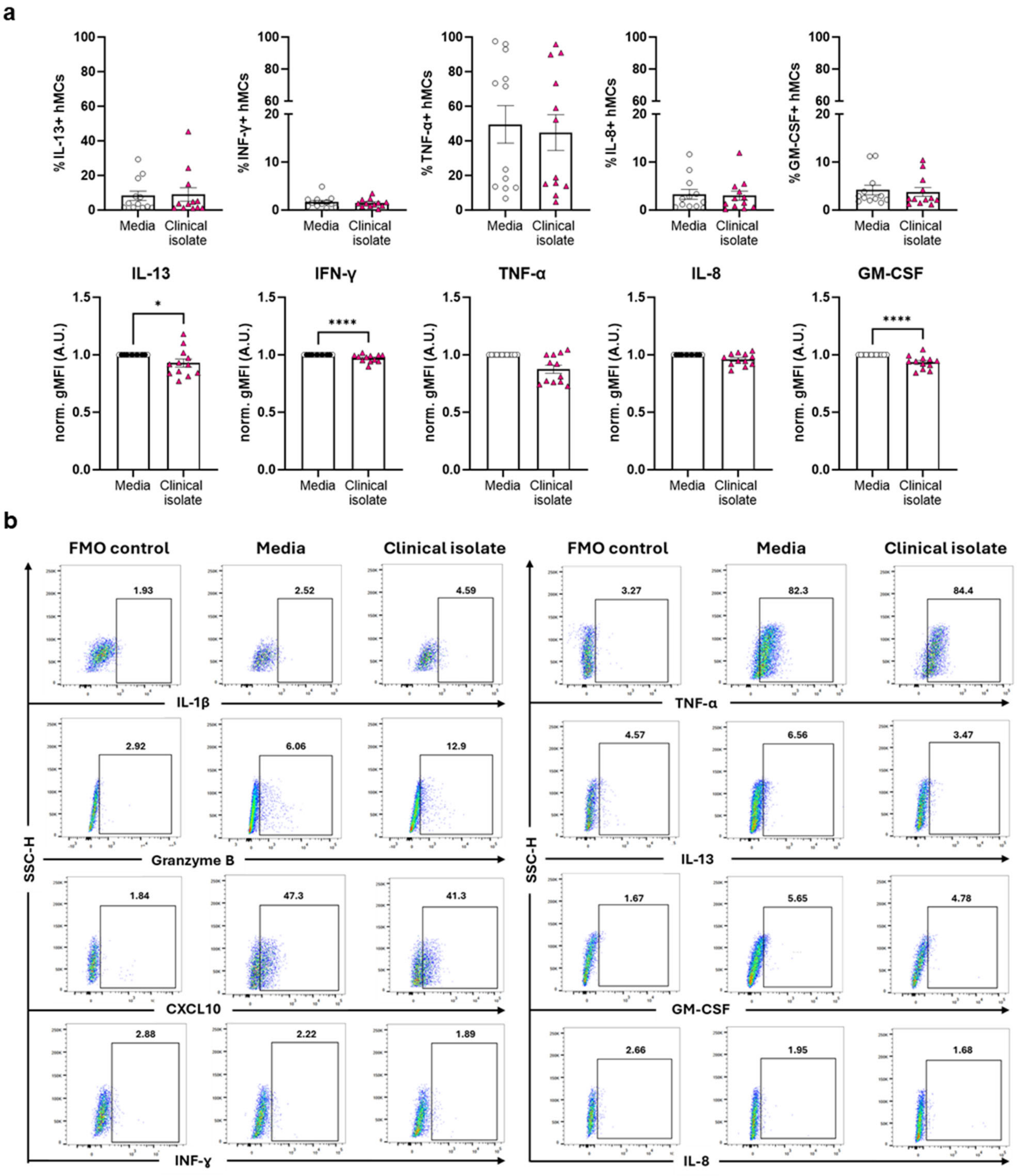

Figure S3. Intracellular mediator expression in hMCs after exposure to *Mab*. hMCs were stimulated for 12 hours with the *Mab* clinical isolate (MOI:10). After stimulation, cells were stained intracellularly with anti-IL-1β, Granzyme B, GM-CSF, IFN-γ, TNF-α, IL-13, CXCL-10, and IL-8 antibodies, and their expression was analysed by flow cytometry. Graphs show the percentage of (**a**) positive cells and of the different cytokines after bacterial stimulation and (**b**) examples of the gating strategies used (representative n=1 donor). Data are from twelve independent experiments and expressed as means ± SEM (n=12). Each symbol represents one donor. Statistical analysis was performed using a paired t-test. A $p<0.05$ was considered statistically significant (****$p< 0.0001$, *$p< 0.05$).

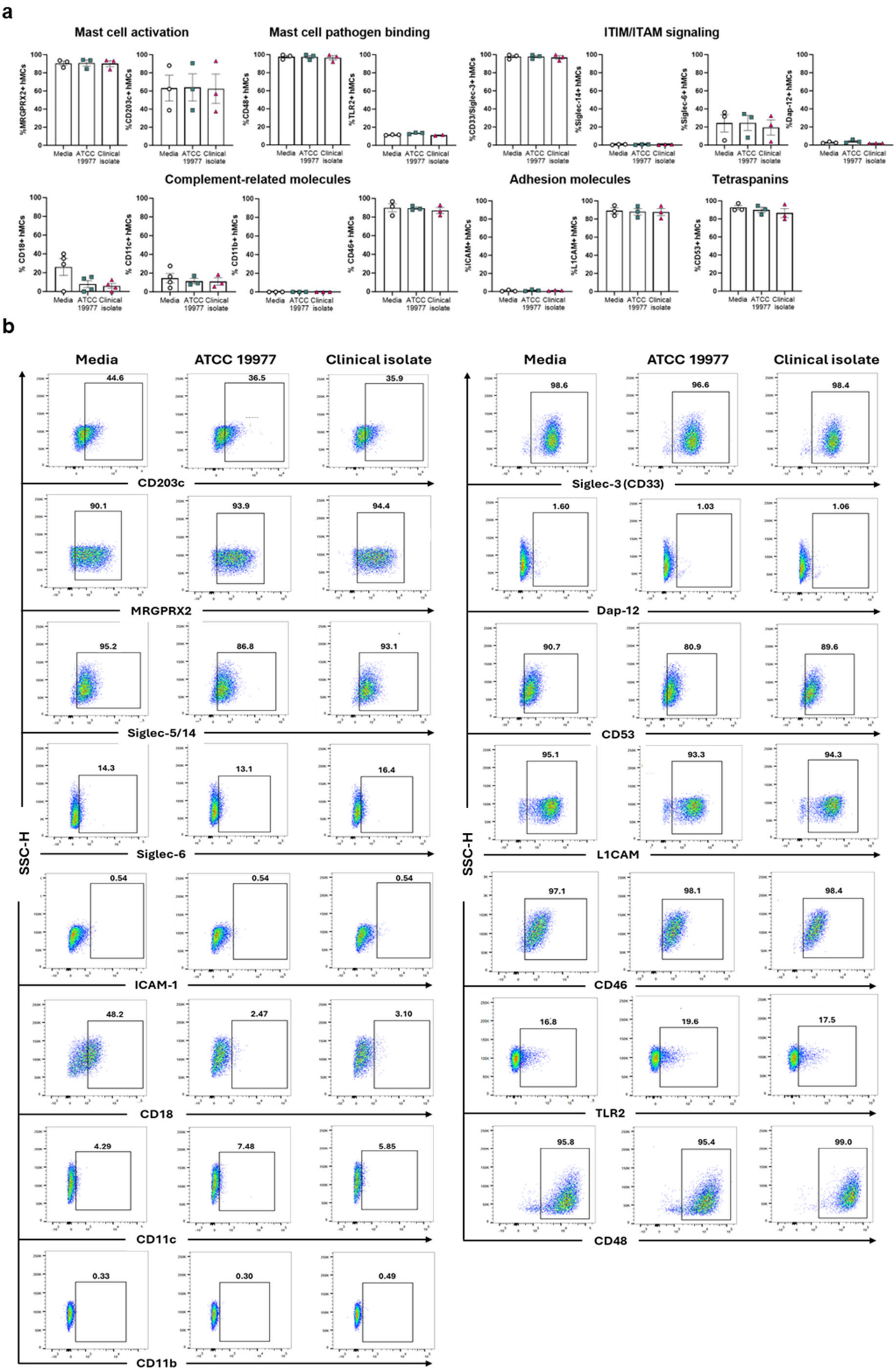
a
Mast cell activation
Mast cell pathogen binding
ITIM/ITAM signaling
%MRGPRX2+ hMCs
%CD203c+ hMCs
%CD48+ hMCs
%TLR2+ hMCs
%CD33/Siglec-3+ hMCs
%Siglec-14+ hMCs
%Siglec-6+ hMCs
%Dap-12+ hMCs
Complement-related molecules
Adhesion molecules
Tetraspanins
% CD18+ hMCs
% CD11c+ hMCs
% CD11b+ hMCs
% CD46+ hMCs
%ICAM+ hMCs
%L1CAM+ hMCs
%CD53+ hMCs
Media
ATCC 19977
Clinical isolate
b
Media
ATCC 19977
Clinical isolate
SSC-H
44.6
36.5
35.9
CD203c
90.1
93.9
94.4
MRGPRX2
95.2
86.8
93.1
Siglec-5/14
14.3
13.1
16.4
Siglec-6
0.54
0.54
0.54
ICAM-1
48.2
2.47
3.10
CD18
4.29
7.48
5.85
CD11c
0.33
0.30
0.49
CD11b
98.6
96.6
98.4
Siglec-3 (CD33)
1.60
1.03
1.06
Dap-12
90.7
80.9
89.6
CD53
95.1
93.3
94.3
L1CAM
97.1
98.1
98.4
CD46
16.8
19.6
17.5
TLR2
95.8
95.4
99.0
CD48

Figure S4. Modulation of receptor expression in hMCs after exposure to *Mab*. hMCs were stimulated for 2 hours with *Mab* ATCC 19977 or clinical strain (MOI:10). After stimulation, cells were stained with anti-MRGPRX2, CD203c, CD48, TLR2, Siglec-3, Siglec-14, Siglec-6, Dap-12, CD53, CD46, ICAM-1, L1-CAM, CD18, CD11c, and CD11b antibodies and their surface expression analysed by flow cytometry. Graphs show (**a**) the percentage of positive cells for each marker after bacterial stimulation and (**b**) examples of the chosen gating strategies for the tested markers (representative n=1). Data are from three independent experiments and expressed as means ± SEM, (n=3). Each symbol represents one donor. Statistical analysis was performed using one-way ANOVA with Tukey's multiple comparisons test. A $p < 0.05$ was considered statistically significant.

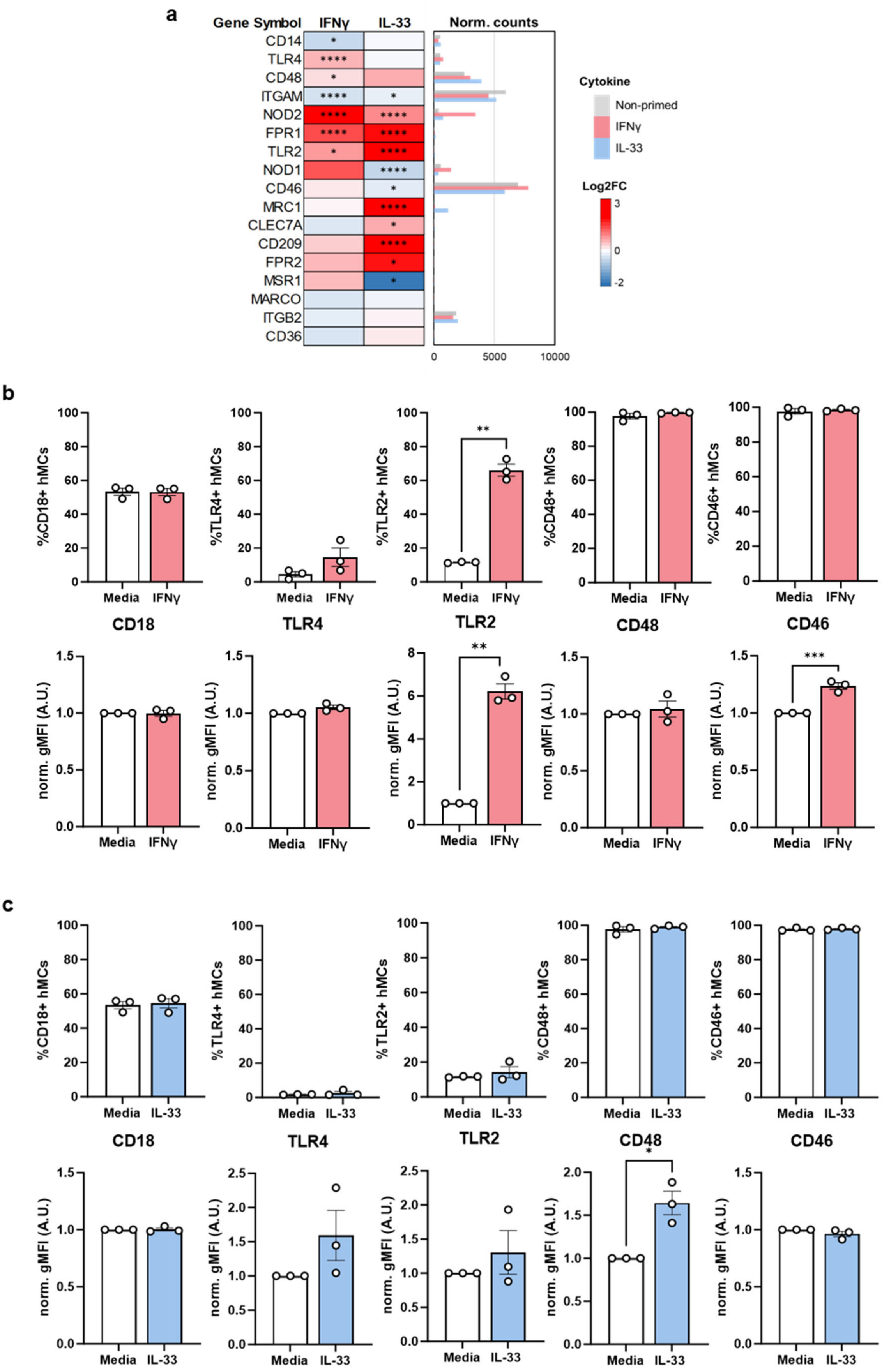
a
Gene Symbol
IFNγ
IL-33
Norm. counts
CD14
TLR4
CD48
ITGAM
NOD2
FPR1
TLR2
NOD1
CD46
MRC1
CLEC7A
CD209
FPR2
MSR1
MARCO
ITGB2
CD36
0
5000
10000
Cytokine
Non-primed
IFNγ
IL-33
Log2FC
3
0
-2
b
%CD18+ hMCs
%TLR4+ hMCs
%TLR2+ hMCs
%CD48+ hMCs
%CD46+ hMCs
Media
IFNγ
CD18
TLR4
TLR2
CD48
CD46
norm. gMFI (A.U.)
c
% CD18+ hMCs
% TLR4+ hMCs
% TLR2+ hMCs
% CD48+ hMCs
% CD46+ hMCs
Media
IL-33

Figure S5. IL-33 and IFN-ɣ promote the expression of pathogen-binding receptor genes on MCs. (**a**) hMCs obtained from 4-6 donors were treated for 24 hours with IL-33, IFN-ɣ (50 ng/mL), or left unstimulated in media II (control). After incubation, mRNA was extracted, and next-generation bulk RNA sequencing was performed. Transcriptomics analysis of selected genes was performed. Log2 fold changes were plotted (FDR-adjusted $p < 0.05$) using Prism 10, and unstimulated mean normalised gene counts of inhibitory receptors expressed by MCs were obtained using DESeq2's normcounts function and plotted in Prism 10. A $p < 0.05$ was considered statistically significant (****$p < 0.0001$, ***$p < 0.001$, **$p < 0.01$). The surface expression of CD48, TLR2, TLR4, CD18, and CD46 was analysed by flow cytometry. Graphs show the percentage of positive cells and the geometric mean fluorescence intensity (normalised by the non-primed media control) after priming with (**b**) IFN-ɣ or (**c**) IL-33 primed-hMCs. Data are from three experiments and expressed as means ± SEM, (n=3). Each symbol represents one donor. Statistical analysis was performed using a paired t-test (%) or the Mann-Whitney test (gMFI). A $p < 0.05$ was considered statistically significant (***$p < 0.001$, **$p < 0.01$, *$p < 0.05$).

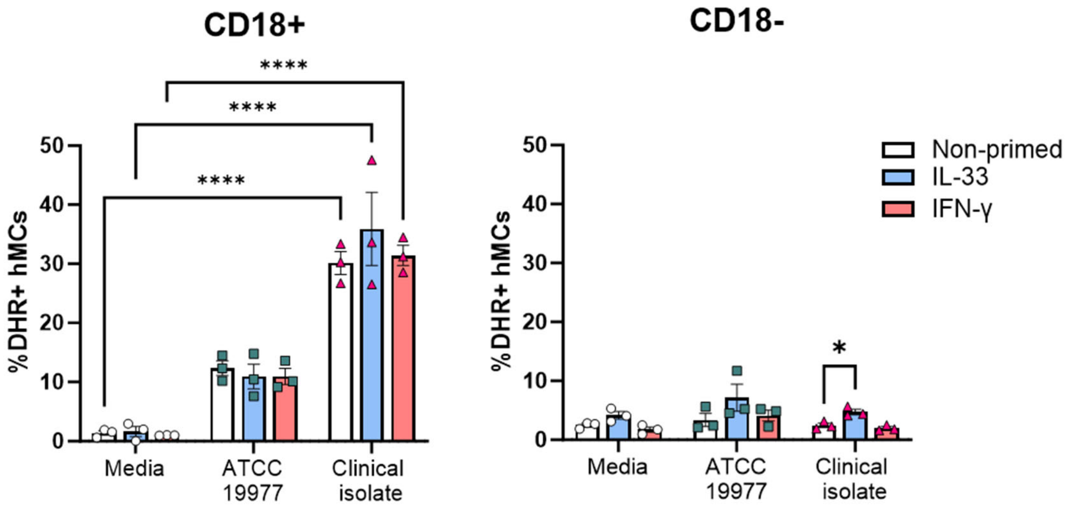

Figure S6. ROS production upon exposure to *Mab* is not affected by IL-33 or IFN-ɣ priming. hMCs were primed with IL-33, IFN-ɣ (50 ng/mL), or media overnight, washed, and pre-loaded with 5 μM DHR dye. Subsequently, cells were stimulated for 2 hours with *Mab* ATCC 19977 or clinical isolate (MOI:10). After stimulation, ROS production was quantified as the percentage of DHR+ cells by flow cytometry. Data are from four independent experiments and are expressed as means ± SEM (n=4). Statistical analysis was performed using two-way ANOVA with Tukey's multiple comparisons test. A $p < 0.05$ was considered statistically significant (****$p < 0.0001$, *$p < 0.05$).

Supplementary Videos

Video S1. Representative live-cell imaging video showing WGA-labelled hMCs (red) exposed to CFSE-labelled *Mab* ATCC 19977 (green) at an MOI of 10. Fluorescence microscopy was performed for 30 min to quantify hMC-*Mab* interactions**.**

Video S2. Representative live-cell imaging video showing WGA-labelled hMCs (red) exposed to CFSE-labelled *Mab* clinical isolate (green) at an MOI of 10. Fluorescence microscopy was performed for 30 min to quantify hMC-*Mab* interactions**.**

Video S3. Representative live-cell imaging video showing WGA-labelled hMCs (red) pre-incubated for 1 h with anti-CD18 blocking antibody before challenge with CFSE-labelled *Mab* clinical isolate (green) (MOI:10). Fluorescence microscopy was performed for 30 min to quantify hMC-*Mab* interactions**.**

Video S4. Representative live-cell imaging video showing WGA-labelled hMCs (red) pre-incubated for 1 h with anti-CD46 blocking antibody before challenge with CFSE-labelled *Mab* clinical isolate (green) (MOI:10). Fluorescence microscopy was performed for 30 min to quantify hMC-*Mab* interactions**.**

Video S5. Representative live-cell imaging video showing WGA-labelled hMCs (red) pre-incubated for 1 h with anti-CD48 blocking antibody before challenge with CFSE-labelled *Mab* clinical isolate (green) (MOI:10). Fluorescence microscopy was performed for 30 min to quantify hMC-*Mab* interactions**.**

Video S6. Representative live-cell imaging video showing WGA-labelled hMCs (red) pre-incubated for 1 h with isotype antibody before challenge with CFSE-labelled *Mab* clinical isolate (green) (MOI:10). Fluorescence microscopy was performed for 30 min to quantify hMC-*Mab* interactions**.**
